# Discoidin Domain Receptors, DDR1b and DDR2, Promote Tumour Growth within Collagen but DDR1b Suppresses Experimental Lung Metastasis in HT1080 Xenografts

**DOI:** 10.1038/s41598-020-59028-w

**Published:** 2020-02-11

**Authors:** Benjamin Wasinski, Anjum Sohail, R. Daniel Bonfil, Seongho Kim, Allen Saliganan, Lisa Polin, Mohamad Bouhamdan, Hyeong-Reh C. Kim, Marco Prunotto, Rafael Fridman

**Affiliations:** 1 Department of Pathology, Wayne State University School of Medicine and Karmanos Cancer Institute, Detroit, MI 48201 USA; 2 Department of Urology, Wayne State University School of Medicine and Karmanos Cancer Institute, Detroit, MI 48201 USA; 3 Department of Oncology, Wayne State University School of Medicine and Karmanos Cancer Institute, Detroit, MI 48201 USA; 4 Hoffmann-La Roche, Basel, Switzerland; 5 School of Pharmaceutical Sciences, Geneva, Switzerland; 6 Present Address: Department of Pathology, College of Medical Sciences and Dr. Kiran C. Patel College of Allopathic Medicine, Nova Southeastern University, Fort Lauderdale, FL 33328–2018 USA

**Keywords:** Cancer microenvironment, Cell signalling

## Abstract

The Discoidin Domain Receptors (DDRs) constitute a unique set of receptor tyrosine kinases that signal in response to collagen. Using an inducible expression system in human HT1080 fibrosarcoma cells, we investigated the role of DDR1b and DDR2 on primary tumour growth and experimental lung metastases. Neither DDR1b nor DDR2 expression altered tumour growth at the primary site. However, implantation of DDR1b- or DDR2-expressing HT1080 cells with collagen I significantly accelerated tumour growth rate, an effect that could not be observed with collagen I in the absence of DDR induction. Interestingly, DDR1b, but not DDR2, completely hindered the ability of HT1080 cells to form lung colonies after intravenous inoculation, suggesting a differential role for DDR1b in primary tumour growth and lung colonization. Analyses of tumour extracts revealed specific alterations in Hippo pathway core components, as a function of DDR and collagen expression, that were associated with stimulation of tumour growth by DDRs and collagen I. Collectively, these findings identified divergent effects of DDRs on primary tumour growth and experimental lung metastasis in the HT1080 xenograft model and highlight the critical role of fibrillar collagen and DDRs in supporting the growth of tumours thriving within a collagen-rich stroma.

## Introduction

The DDRs constitute a unique subfamily of receptor tyrosine kinases (RTKs) that signal in response to collagens, and therefore they represent the only RTKs that are directly activated upon contact of cells with their collagenous matrix^1–6^. The DDR subfamily comprises two kinases: DDR1 and DDR2^1,2,7^. The DDR1 subfamily includes six isoforms generated by alternative splicing, while there is only one form of DDR2. Among the DDR1 isoforms, DDR1a, DDR1b, and DDR1c are full length, highly homologous receptors, with the only structural difference being the presence of 37 additional residues, including two additional tyrosine residues, within the intracellular region of DDR1b and DDR1c, suggesting that these species may activate different downstream signalling pathways^1^. DDR1d and DDR1e are truncated, kinase-deficient receptors of unknown function^8^. DDR1f contains a full kinase domain but lacks most of the extracellular domains^9^. Structurally, the DDRs are type I transmembrane multidomain glycoproteins. The extracellular portion of both DDRs contain the Discoidin Domain, a unique structural domain that comprises the collagen binding sites^10^. Like all RTKs, the DDRs contain a kinase domain in the intracellular portion of the receptor that is activated upon collagen binding resulting in receptor autophosphorylation at multiple tyrosine residues present within the intracellular region^1,2,6^. Both receptors recognize collagens as their ligands with some differences: the functional DDR1 isoforms bind and signal in response to both fibrillar and non-fibrillar collagens whereas DDR2 is mostly activated by fibrillar collagens^2^. DDR1 is usually expressed in epithelial tissues while DDR2 is mostly expressed in mesenchymal cells. However, both epithelial- and mesenchymal-derived cells have been shown to express either one or both receptors in physiological and pathological conditions^11–21^. Consistently, both DDR1 and DDR2 are expressed in cancers of epithelial^18,19,22–30^ and mesenchymal^17,31,32^ origin. Functionally, DDRs are thought to mediate tumour cell-collagen interactions at various stages of cancer progression, where they initiate signal transduction pathways that regulate epithelial-to-mesenchymal transition (EMT), cell proliferation and survival, and metastatic dissemination^3,33^. Indeed, in mouse models of cancer, DDR1 and DDR2 were shown to play a role in tumour growth and/or metastases of epithelial^21,23,29,30,34–45^ and mesenchymal^32^ cancers. Thus, significant experimental evidence supports a role for DDRs in promotion of cancer progression.

In spite of the accumulating evidence and considering that both DDRs can be expressed by the same tumour type, a comparison of DDRs’ action in tumour growth and metastases within the same cellular background has not been conducted. To address this limitation and to gain more insight into the roles of DDRs in malignancy, we set to investigate the contribution of DDR1b and DDR2 to the tumorigenic and lung colonizing abilities of human fibrosarcoma HT1080 cells. These malignant cells display a consistent ability to form rapidly proliferating tumours when inoculated subcutaneously (s.c.) into immunodeficient mice. Although s.c. HT1080 tumours do not generate spontaneous metastases, intravenous inoculation of HT1080 cells yields numerous tumour colonies in the lung parenchyma, making these cells a useful tool to investigate late steps of the metastatic cascade. Thus, the HT1080 cell system is a dependable xenograft model to elucidate the contribution of factors to tumour growth and experimental lung metastases. Importantly, like other mesenchymal cell lines and tumours^17,31,32,46^, HT1080 cells express DDR1 and DDR2^26,47–50^. *In vivo*, fibrosarcomas are characterized by an abundant collagen matrix, which has been suggested to promote malignancy^51,52^. Thus, DDR-collagen interactions may play a role in fibrosarcoma progression. In this study, we used a Tet-Off^®^ system to regulate the expression of DDR1b or DDR2 in HT1080 cells, as previously reported^47^. We focused on the DDR1b isoform because it is a full-length functional receptor species that is ubiquitously expressed in mammalian cells^1,3^. Here we report that while both DDR1b and DDR2 significantly restrict HT1080 *in vitro* cell proliferation in 2D and 3D collagen I matrices, *in vivo* DDRs accelerate tumour growth only when the cells are implanted within a collagen I (COL1) gel. DDR/COL1-enhanced tumour growth was associated with specific alterations in the Hippo pathway, a major signalling tumour suppressor pathway regulated in part by extracellular matrix (ECM) components^53,54^. We also report that DDR1b, but not DDR2, expression potently suppressed the ability of HT1080 cells to form lung colonies after intravenous inoculation. Thus, DDRs elicit divergent effects on tumour cell malignancy in a context-dependent manner.

## Materials and Methods

### Cell Culture

Human HT1080 fibrosarcoma cells^55^ were obtained from the American Type Culture Collection (ATCC, Rockville, MD). The cells were routinely cultured in DMEM (Gibco, Waltham, MA) supplemented with 1% penicillin, 1% streptomycin, and 8% tetracycline-free foetal bovine serum (FBS) from Takara (Japan; Cat# 631106). Other human cell lines used in this study are described in the Supplemental Information (Supplementary Fig. 3).

### Generation of HT1080 cells with inducible expression of DDR1b or DDR2

Tet-Off^®^ inducible DDR1b- or DDR2-expressing human HT1080 fibrosarcoma cells were generated as described previously^56,57^. An individual clone of DDR1b- or DDR2-expressing cells, referred to as HT-DDR1b and HT-DDR2 cells, respectively, was selected for the studies conducted here. The engineered HT1080 cell lines were certified by the Wayne State University’s Biobanking and Correlative Sciences Core and were found to exhibit a 100% pass-match with the HT1080 cell line.

### Antibodies, extracellular matrix proteins, enzymes, and chemicals

A complete and detailed list of the polyclonal and monoclonal antibodies used in this study is provided in Supplementary Table 2. Doxycycline (DOX) hyclate was purchased from Sigma (St. Louis, MO; Cat #D9891). Rat-tail COL1 (regular and high concentration) was purchased from Discovery Labware Inc., Corning™ (Bedford, MA; Cat # 354236, regular; and # 354249, high concentration). Mouse collagen IV was purchased from Corning™ (Cat # 354233). Matrigel (Cultrex^®^) was purchased from Trevigen (Gaithersburg, MD; Cat # 3444-005-01). Bacterial collagenase was purchased from Sigma (Cat# C9263). Trypsin-EDTA was purchased from Gibco (Cat # 25200).

### DOX treatment and regulation of DDR expression

To repress DDR expression, the HT-DDR1b and HT-DDR2 cells were incubated in complete media supplemented with 50 μg/ml (final concentration) of DOX. To induce DDR expression *in vitro*, HT-DDR1b and HT-DDR2 cells were washed (2×) with phosphate buffered saline (PBS) and cultured in the absence of DOX in complete media. After three days, the media were aspirated, and the cells were processed for various analyses or biological assays. Some assays were conducted with or without DOX supplementation to maintain DDR repression or induction, respectively, during the assay conditions

### Preparation of fibrillar COL1 for 2D and 3D conditions

Fibrillar rat-tail COL1 was prepared according to the manufacturer’s protocol (Corning™).

### Collagen-induced DDR activation

HT-DDR1b and HT-DDR2 cells cultured for three days with or without DOX, as described above, were washed (2×) with PBS followed by addition of serum-free media with or without DOX (50 μg/ml final concentration). After 18 h at 37 °C, HT-DDR1b cells received 20 μg/ml (final concentration) of either rat-tail COL1 or mouse collagen IV (COL4) diluted in 20 mM acetic acid (COL1) or 50 mM HCl (COL4), whereas HT-DDR2 cells received same amounts of rat-tail COL1. Control cells received equal volume of acetic acid or HCl. After various incubation times at 37 °C, the cells were washed twice with cold PBS, and lysed for immunoblot analyses.

### Preparation of cell lysates and tumour extracts

Cultured cells were lysed in RIPA buffer (50 mM Tris-HCl, pH 7.4, 150 mM NaCl, 1% NP-40, 0.25% sodium deoxycholate and 1 mM EDTA) supplemented with protease inhibitors (Roche, complete, Mini, EDTA-free) and 10 mM sodium fluoride and 1 mM sodium orthovanadate. The cell lysates were cleared by centrifugation at 13,000 g at 4 °C for 15 min, and the protein concentration was determined using the BCA kit from Pierce (Waltham, MA; Cat # 23227). For preparation of tumour extracts, s.c. tumours were cut with sterile scissors into small pieces. The pieces were weighed, and then placed in a 1.5 ml microcentrifuge tube followed by addition of RIPA buffer (10 vol/weight). The tissues were then homogenised by manual tissue grinding. After a 2-h incubation at 4 °C with gentle rotation, the tumour extracts where centrifuged at 13,000 rpm, the supernatants were transferred to a fresh tube, and then snap-frozen in liquid nitrogen. Cell lysates and tumour extracts were kept at −80 °C until further use.

### Immunoblot analyses

Expression and activation of DDRs in the HT1080 cells and in tumour extracts were analysed by immunoblot analyses using specific antibodies recognizing total or phosphorylated receptors. To this end, equal amounts of protein from each lysate isolated from cultured cells or s.c. tumours were resolved by reducing 7.5% or 10% SDS-PAGE. Proteins were then transferred to a nitrocellulose membrane using conventional methods. The blots were probed with specific antibodies to the proteins of interest (antibodies described in Supplementary Table 2) and then the same blot was reprobed for β-actin, as a loading control. In the case of phosphorylated proteins (DDRs, YAP1), blots were first probed with antibodies to the phosphorylated form and then stripped and reprobed with antibodies to the total (unphosphorylated) protein. Then, the blots were probed for β-actin. Antigen/antibody complexes were visualized using the SuperSignal West Pico Plus and/or the SuperSignal West Femto Maximum Sensitivity Substrate from ThermoFisher Scientific (Rockford IL; Cat # 34580 and 34095, respectively). Antigen levels in the immunoblots were quantified using ImageJ software program (NIH, USA) by measuring each lane for pixel intensity and normalizing to β-actin levels. The extent of phosphorylated protein was obtained by taking a ratio of phosphorylated/unphosphorylated (total) protein bands in the same lane. Otherwise indicated in the Figure legend, comparisons between samples were only made between samples run on the same gel. Whole blots are presented in Supplemental Figures.

### *In vitro* cell proliferation assays in 2D and 3D COL1 conditions

HT-DDR1b and HT-DDR2 cells were incubated with or without DOX three days prior to seeding of the cells for the growth assay to repress or induce DDR expression. The cells were then harvested and seeded atop a thin layer of fibrillar COL1 (2D) or embedded within a COL1 (3D) matrix, in the presence or absence of DOX, in complete media. For 2D conditions, COL1-coated wells were prepared by adding 100 μg/well of fibrillar COL1 into 24-well plates, followed by an incubation at 37 °C, 5% CO_2_ to allow fibrillar collagen formation. Then, 2 × 10^4^ cells/well in complete media were seeded on either on top of the fibrillar COL1 or on uncoated wells, in triplicates. At various time points, the cells were detached with a mixture of trypsin-EDTA and collagenase (10 U/mg of collagen), resuspended in complete media, and then counted with a particle counter (Coulter, Z1 Particle) at settings of 10–30 μm particle size. For 3D conditions, the cells were mixed with a neutralized COL1 solution (2 mg/ml, final concentration), prepared as described above. Eight replicates of the 40 μl cell-COL1 mixtures were then added to a 96-well plate to a final density of 1 × 10^3^ cells per well. The plates were then incubated at 37 °C, 5% CO_2_ to allow COL1 gelling. Next, 100 μl of complete medium with or without DOX were added to each well. Cells were then incubated at 37 °C, 5% CO_2_ for 3 or 11 days with a media change every 2–3 days. Cell growth was determined using the XTT assay (ATCC, Manassas, VA; Cat# 30–1011), as described by the manufacturer.

### *In vitro* tumour cell invasion assays

Invasive ability was evaluated using the spheroid invasion assay^58^ and the Boyden Chamber assay. For the spheroid invasion assay, 2.5 × 10^3^ cells/well were plated in the presence or absence of DOX in ultra-low attachment (ULA) 96-well plates (Corning, Cat # 7007) in replicates of six. The cells were then incubated for 4 days at 37 °C, 5% CO_2_ to allow spheroid formation. On day 4, DOX was removed from a subset of the +DOX group by diluting the DOX 64-fold (see Results section). Then, 100 μl/well of neutralized COL1 (2 mg/ml final concentration) was added to the wells and allowed to polymerize for 1 h at 37 °C, 5% CO_2_. After collagen polymerization, complete media (100 μl/well) was placed on top of the gels. Cultures were photographed at 0, 24, and 48 h time points. Areas of invasion were quantified by tracing the edges of the invasive spheroids and calculating total pixels using ImageJ software. Replicate wells were then averaged and reported as shown in the results section.

Tumour cell invasion was also evaluated using Matrigel, an extract of basement membrane proteins^59^, as a matrix barrier. Corning™ Transwell^®^ culture inserts fitted with a polycarbonate filter (8-μm pore size, 6.5-mm diameter) (Cat # 3422), were coated with Matrigel. Briefly, 50 μl of an ice-cold solution of Matrigel (1 mg/ml) were applied to each filter and allowed to gel at 37 °C for 2 h. HT-DDR1b and HT-DDR2 cells were incubated 3 days in the presence or absence of DOX to repress or induce DDR expression, respectively. The night prior to seeding, the cells were washed (2×) with PBS and incubated in serum free media overnight in the presence or absence of DOX. The following day, 7.5 × 10^4^ cells/filter were plated in triplicate, in serum free medium (100 μl/insert), on the Matrigel-coated inserts with or without DOX supplementation. The inserts were placed on the wells, which were filled with 650 μl/well of medium containing 8% FBS, as attractant, ±DOX supplementation. The plates were placed in an incubator at 37 °C, 5% CO_2_ and the cells were allowed to invade for 8 h before being fixed and stained with Diff-Quick (Siemens, Newark, DE; Cat # B4132-1A). Five pictures (four from upper/lower quadrant corners and one from the center) were taken at 20x magnification of each membrane and cells were counted, averaged, and reported as “Average # of number of cells/field.”

### Subcutaneous tumour growth and experimental lung metastasis assay

All animal procedures were approved by the Institutional Animal and Care Use Committee (IACUC) of Wayne State University (Protocol ID: 16-03-053) prior to experiments. Thus, all animal experiments were performed in accordance with relevant guidelines and regulations. All animal studies were conducted with 4–6-week-old female *SCID/NCr (strain code 561)* mice (Charles River Laboratories Inc., Wilmington, MA). The number of mice utilized in in each group is depicted in Supplementary Table 1. Before tumour cell inoculation, in both tumorigenicity and lung colonization assays, mice were divided in two groups: +DOX and −DOX mice. The +DOX mice were fed (*ad libitum*) a standard diet of irradiated feed supplemented with 625 mg/Kg of doxycycline hyclate (Envigo, Indianapolis, IN; Cat #TD.140263) starting three days prior to tumour cell inoculation. This diet delivers a daily dose of ~2–3 mg of DOX, based on a consumption of 4–5 g/d by a mouse (information provided by the supplier). The −DOX mice were fed the standard diet of irradiated feed. Two days before tumour cell inoculation, a set of tissue culture dishes with HT-DDR1b or HT-DDR2 cells were washed with serum-free medium and incubated in complete medium without DOX to induce DDR expression. Another set of cells was cultured in the presence of DOX to repress DDR expression. On the day of cell inoculation, the cells were harvested and resuspended in serum-free DMEM with or without DOX. For evaluation of tumorigenicity, mice were inoculated subcutaneously (s.c.) with 1 × 10^6^ cells/mouse of HT-DDR1 or HT-DDR2 cells (with or without DOX pre-treatment) in 100 µl of serum-free DMEM. Another group of mice were inoculated with same number of HT1-DDR1b and HT-DDR2 cells (with or without DOX) suspended in a solution of COL1, as follows: a neutralized solution of rat-tail COL1 was prepared as stated above and mixed with a 10x concentration of cells (1 × 10^7^ cells) so that the final concentration of fibrillar COL1 was 2 mg/ml, and the final number of cells was 1 × 10^6^ /mouse, in 100 µl. Tumour sizes were measured at least twice a week with a calliper. The tumour volume was calculated using the formula *V* = (*LW*^2^) × *0.4,* where *V* is the volume (mm^3^); *L*, is the longest diameter (mm) and *W*, is the shortest diameter (mm). Mice were euthanized ~6 weeks after tumour cell inoculation. However, when s.c. tumours became significantly ulcerated before reaching the allowed size (2,000 mm^3^), mice within the same group were euthanized earlier. Tumours were harvested and snap frozen in liquid nitrogen and stored at −80 °C or fixed in 4% paraformaldehyde, until further use.

For experimental lung metastases (lung colonization assay), 1 × 10^6^ of either HT-DDR1b or HT-DDR2 cells (with or without DOX pre-treatment) were inoculated into the tail vein of +DOX or −DOX mice, as described above, in a final volume of 100 µl PBS. Mice were sacrificed ~20 days after tumour cell inoculation or after significant signs of distress. Lungs were harvested, photographed, and then sectioned so that, from each mouse, the entire left lung was placed in 4% paraformaldehyde for histological analyses, and the entire right lung was snap frozen in liquid nitrogen and immediately stored at −80 °C for DNA extraction and *Alu* qPCR analysis.

### qPCR and *Alu* sequence analyses

The sequence of the primers used in this study are described in Supplementary Table 3. For analyses of DDR1 isoforms, total RNA was isolated from HT-DDR1b cells grown in the presence of DOX, as described above. For analyses of CTGF, CYR61, AXL mRNA expression, total RNA was isolated from s.c. tumours. RNA was isolated by TRIzol™ Reagent according to the manufacturer’s protocol and used to generate cDNA using iScript™ reagent (BioRad, Hercules, CA; Cat# 1708840), according to the manufacturer’s protocol. Real time PCR was then performed using the SYBR® Select Master Mix Kit (Applied Biosystems, Cat # 4472908), according to the manufacturer’s protocol. The mRNA signals were normalized using human GAPDH as a reference, and the relative amounts of DDR1 isoforms, CTGF, CYR61, and AXL mRNAs were expressed as the percentage of GAPDH. Analyses of human *Alu* sequences were performed using DNA isolated from the right lungs of mice inoculated intravenously with HT-DDR1b and HT-DDR2 cells, essentially as described^60,61^. Real time PCR was then performed using the SYBR® Select Master Mix Kit, according to the manufacturer’s protocol.

### Statistical analyses and data presentation

Data from *in vitro* assays and immunoblots were statistically compared by either unpaired *t*-test with log-transformation or nonparametric Mann-Whitney U test. All experiments were repeated at least three times. For the mice studies, tumour growth rates across time were compared using a linear mixed-effects model with a change of point model in order to account for the early stage without tumour after data were log-transformed^62^. The linear mixed-effects model was allowed for subject-specific baseline tumour size and tumour growth rate considering the correlation between time-dependent observations within the same subject. To assess the tumour size at the last time point, either unpaired t-test with log-transformation or nonparametric Mann-Whitney U test was used. When the last observation time points were different between two groups, the tumour growth rate and size comparisons were performed using the data until the minimum last observation time points. Tumour latency, until detection of a tumour of a defined size (1,000 mm^3^), was summarized by Kaplan-Meier estimates and compared by a log-rank test. Note: although all data sets were obtained under the same experimental conditions and repeated multiple times, in some instances the same set of data (from a specific experimental group) were plotted in a different figure to compare it to a different group. This was done with the goal of reducing figure crowding and to better highlight the differences among multiple experimental groups. When the data have been replotted, this is indicated in the pertinent Figure legends. All statistical comparisons were performed at a 2-sided 5% significance level.

## Results

### Expression and activation of DDR1b and DDR2 in HT1080 cells by the Tet-Off^®^ system

To express recombinant DDRs using the Tet-Off^®^ expression system, we utilized human fibrosarcoma HT1080 cells. Cells were engineered to express DDR1b or DDR2 upon withdrawal of DOX. DDR1 has five isoforms but we chose to express the b isoform because this is a full-length, kinase functional DDR1 receptor that is also ubiquitously expressed^1^. The engineered HT1080 cells, generated as described in the Methods section, were analysed for endogenous levels of DDR protein and for profile of endogenous DDR1 isoforms. To this end, HT-DDR1b and HT-DDR2 cells, cultured in the presence of DOX to repress ectopic DDR expression, were processed for immunoblot and qPCR analyses. In agreement with previous studies using HT1080 cells^26,49^, HT-DDR1b and HT-DDR2 cells express endogenous DDR1 and DDR2 (Supplementary Fig. 1A,B, respectively). qPCR analyses demonstrated that the major endogenous DDR1 isoforms expressed by DOX-treated HT-DDR1b cells are DDR1a and DDR1b and relatively lower levels of DDRc, while isoforms d and e transcripts were not detected, under the experimental conditions (Supplementary Fig. 1C).

**Figure 1:**
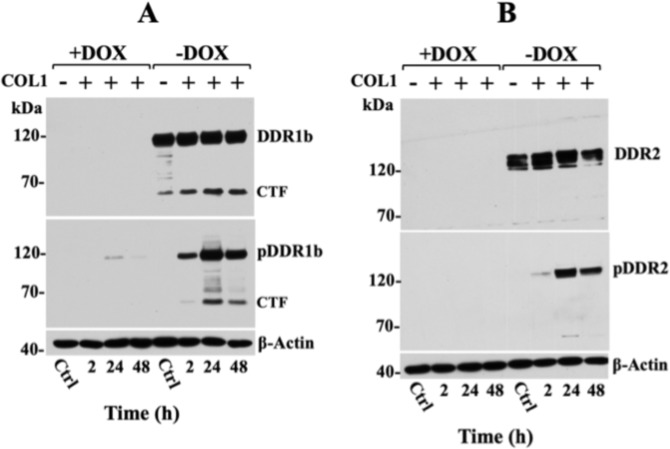
Doxycycline regulated expression of recombinant DDR1b and DDR2 in HT1080 cells. HT-DDR1b (**A**) and HT-DDR2 (**B**) cells processed to express or repress recombinant DDRs, as described in the Methods section, were incubated in serum-free media supplemented with or without 20 μg/ml of COL1 for various times at 37 °C. At the end of the incubation period, the cells were lysed in RIPA buffer and equal protein amounts per lane (30 µg) were resolved by reducing SDS-PAGE followed by immunoblot analyses using antibodies to phosphorylated DDR1b (Y513) (**A**, middle panel) or DDR2 (Y740) (**B**, middle panel). The same blots were then stripped and reprobed with antibodies against total DDRs (upper panels), and against β-actin, as loading control (lower panels). Control (Ctrl.): cells (harvested at 48 h), which were incubated with or without DOX but not stimulated with COL1. CTF, C-terminal fragment of DDR1b; pCTF, pDDR1b, and pDDR2 refers to the phosphorylated protein. Full-length blots are presented in Supplementary Fig. 9.

Next, we examined the regulation of recombinant DDRs in HT-DDR1b and HT-DDR2 cells as a function of DOX. As shown in Fig. 1, immunoblot analyses of HT-DDR1b and HT-DDR2 lysates confirmed DOX-mediated regulation of recombinant DDR1b (Fig. 1A, ~120 kDa) and DDR2 (Fig. 1B, ~130 kDa), as expected. The DDR1b antibodies also displayed immunoreactivity with a ~57-kDa protein, which represents proteolytically cleaved DDR1b comprising the entire intracellular domain^27,63–65^. This form is referred to as the C-terminal fragment (CTF) of DDR1. Treatment with COL1 activated both DDR1b and DDR2 (Fig. 1A,B, middle panel). The CTF of DDR1b is also phosphorylated, as it contains the pY513 site detected by the pDDR1b Ab used here. Exposure of HT-DDR1b cells to basement membrane collagen IV (COL4), a ligand of DDR1 but not DDR2^1^, resulted in activation of DDR1b, as expected (Supplementary Fig. 2). To estimate the relative levels of recombinant DDR expression by the Tet-Off^®^ system, at the conditions used throughout this study, we compared them with those found in a variety of established human cancer cell lines with endogenous DDR expression (Supplementary Fig. 3). These immunoblot analyses revealed that the levels of recombinant DDR1b in HT-DDR1b cells were ~2-folds higher than those detected in BPH1 and HCC1806 cells, and 4-folds higher than those detected in CFPAC-1 and T47D cells (Supplementary Fig. 3A). Recombinant DDR2 levels in HT-DDR2 cells were ~2.5-folds higher than those found in SUM1315 cells (Supplementary Fig. 3B). Taken together, these studies demonstrate the inducible regulation of DDRs by DOX and their collagen-initiated activation in the HT1080 cell system.

### DDR expression in HT1080 cells has no effect on tumour growth *in vivo* or cell proliferation *in vitro*

**Figure 2:**
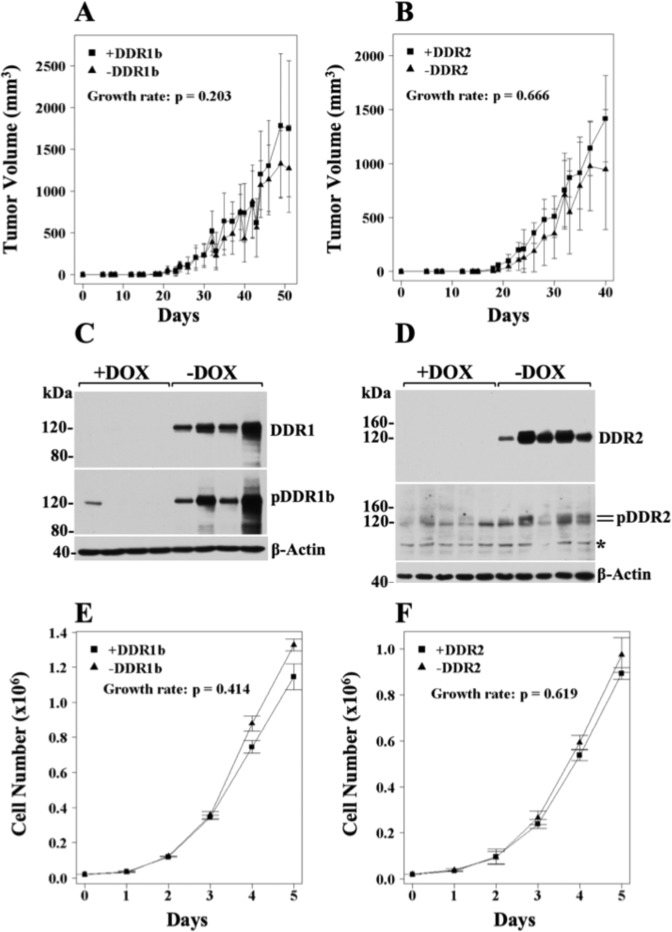
DDRs do not alter tumour growth rate of HT1080 cells *in vivo* or *in vitro*. (**A**,**B**) Tumour volumes of ±DDR tumours as a function of time. HT-DDR1b (**A**) and HT-DDR2 (**B**) cells were incubated for two days with or without DOX to repress or induce DDRs, respectively. Then, 1 × 10^6^ cells/mouse were inoculated s.c. into mice that were fed a regular or a DOX-supplemented diet. Number of mice for each group is provided in Supplementary Table 1. Tumours were measured every 2–3 days, and tumour volumes were calculated. Tumour growth rates were determined, as described in the Methods section. (**C**,**D**) Expression and activation of DDRs in ±DDR tumour extracts. Tumour extracts from representative mice (n = 4 for HT-DDR1b ± DOX and n = 5 for HT-DDR2 ± DOX) in RIPA buffer were resolved by reducing SDS-PAGE followed by immunoblot analyses using antibodies against phosphorylated DDR1b (Y513) (**C**, middle panel) or DDR2 (Y740) (**D**, middle panel). The same blots were then stripped and reprobed with antibodies to total DDRs (upper panels), and then reprobed for β-actin, as a loading control (lower panels). Asterisk indicates a non-specific band. Full-length blots are presented in Supplementary Fig. 10. (**E**,**F**) *In vitro* cell proliferation of ±DDR-expressing HT1080 cells on plastic. HT-DDR1b (**E**) and HT-DDR2 (**F**) cells were incubated with or without DOX three days and then seeded on plastic in 24-well plates (2 × 10^4^ cells/well) in triplicate in the presence or absence of DOX, in complete media. At various time points, the cells were harvested and counted with a Coulter counter. Growth rate were calculated as described in Methods. Results represent the average of three independent experiments.

The HT1080 system is a dependable xenograft model to investigate many of the molecular and cellular processes involved in tumour growth due to the consistent and efficient ability of these cells to form s.c. tumours in immunodeficient mice. We therefore set to investigate the effects of DDRs on HT1080 tumour growth upon s.c. inoculation, as described in the Methods section. We found that neither DDR1b nor DDR2 overexpression (−DOX diet) had an impact on HT1080 tumour growth rate when compared with tumours with repressed DDR expression (+DOX diet) (Fig. 2A,B). Note that Figures of tumour volumes indicate only differences (*p* values) in tumour growth rate. Tumour size and latency *p* values are provided in Supplementary Table 1. Immunoblot analyses of tumour extracts confirmed DOX-dependent regulation of recombinant DDRs *in vivo* (Fig. 2C,D). Analyses of DDR phosphorylation status revealed that both recombinant receptors were phosphorylated (Fig. 2C,D, middle panels), likely by naturally produced collagen within the tumour microenvironment. Thus, interestingly, receptor phosphorylation was not associated with changes in tumour growth rate. Next, we sought to evaluate the effects of DDRs on HT1080 cell proliferation *in vitro*, on cells seeded on plastic dishes. These studies showed that neither DDR1b nor DDR2 expression altered HT1080 cell growth when compared to cells with repressed DDR expression (Fig. 2E,F, respectively). Together, these results demonstrated that, under these conditions, ectopic DDR expression in HT1080 cells had no impact on tumour growth, in agreement with previous studies showing a lack of effect on cell proliferation by DDRs in various cell systems^43,56,66–68^.

### Divergent effects of DDRs and COL1 in HT1080 cell growth *in vivo* and *in vitro*

**Figure 3:**
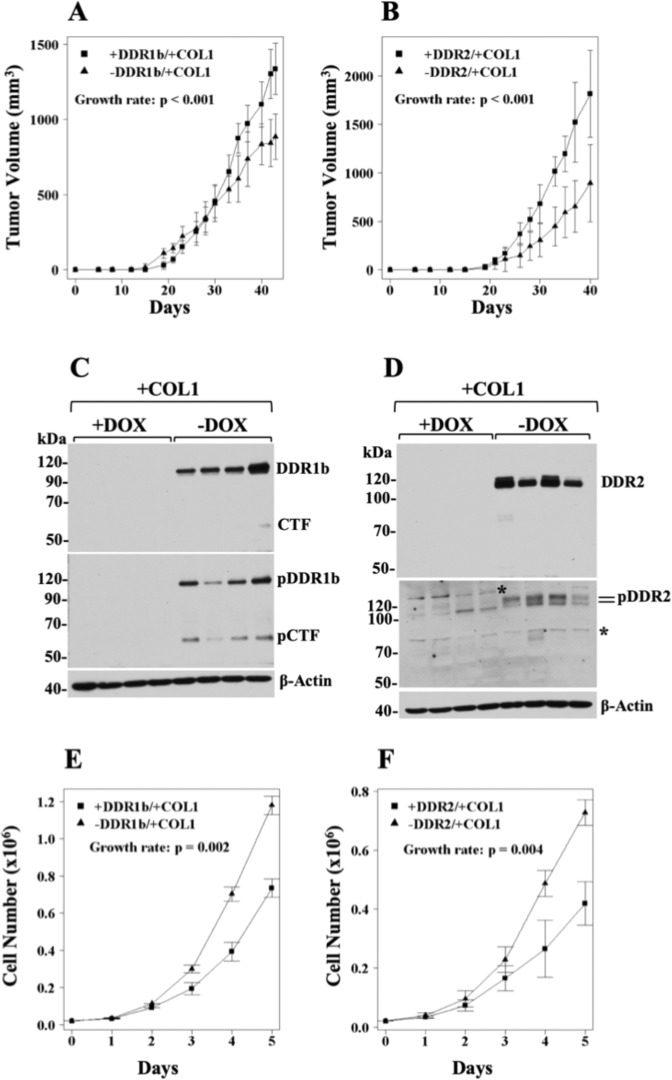
DDRs and COL1 promote the growth rate of HT1080 cells *in vivo* but not *in vitro*. (A,B) Volumes of ±DDR/+COL1 tumours as a function of time. HT-DDR1b (**A**) and HT-DDR2 (**B**) cells were incubated for two days with or without DOX to repress or induce DDR expression, respectively. Then, the cells were harvested and mixed with an ice-cold solution of COL1 (2 mg/ml, final concentration), as described in the Methods section. Mice fed a regular or a DOX-supplemented diet were inoculated s.c. with 1 × 10^6^ cells/mouse in 100 μl of the COL1/cell suspension. Number of mice for each group is provided in Supplementary Table 1. Tumours were measured every 2–3 days, and tumour volumes were calculated. Tumour growth rates were determined, as described in the Methods section. (**C**,**D**) Expression and activation of DDRs in ±DDR/+COL1 tumour extracts. Tumour extracts derived from representative mice harbouring either HT-DDR1b (**C**) and HT-DDR2 (**D**) ±DOX tumours (n = 4 in each group) were resolved by reducing SDS-PAGE followed by immunoblot analyses using antibodies against phosphorylated DDR1b (Y513) (**C**, middle panel) or DDR2 (Y740) (**D**, middle panel). The blots were then stripped and reprobed with antibodies against total DDRs (upper panels), and then reprobed for β-actin (lower panels). pCTF, phosphorylated C-terminal fragment of DDR1b. Asterisk indicates a non-specific band. Full-length blots are presented in Supplementary Fig. 11. (**E**,**F**) *In vitro* cell proliferation of ±DDR -expressing HT1080 cells in 2D COL1. HT-DDR1b (**E**) and HT-DDR2 (**F**) cells were incubated with or without DOX for three days, and then 2 × 10^4^ cells/well were seeded on 24-well plates coated with fibrillar COL1 (100 μg/well), in triplicates, in complete media. At various time points, the cells were detached and counted with a Coulter counter. Results represent the average of three independent experiments.

Engraftment of tumours within ECM components, in particular within Matrigel, a collagen IV- and laminin-rich matrix, has been shown to promote the survival and growth of established cancer cell lines, including HT1080 cells, cancer stem cells, and patient-derived tumours in mice^69–71^, consistent with the critical role of the ECM in tumorigenicity^72^. Because DDRs had no effect on s.c. tumour growth (Fig. 2A,B), we asked whether co-implantation of HT1080 cells with COL1 would uncover a pro-tumorigenic activity of DDRs. To address this hypothesis, we chose COL1, as a matrix scaffold, because it is a ligand for both DDR1b and DDR2^1^, and fibrosarcomas are known to thrive within a fibrillar collagen-rich environment^51^. Moreover, COL1 is the major component of the pro-fibrotic stroma of many cancer types^73^. As depicted in Fig. 3A,B (and in Supplementary Table 1), co-implantation of DDR1b- or DDR2-expressing HT1080 cells with COL1, resulted in the formation of tumours that grew significantly faster than those generated by cells without DDR1b or DDR2 induction (*p* < *0.001* for both receptors), demonstrating a pro-tumorigenic effect of DDRs and COL1 on HT1080 xenografts. Immunoblot analyses of tumour extracts confirmed DDR expression and activation only in tumours of mice that were fed a diet without DOX, as expected (Fig. 3C,D). The pDDR1b blot showed presence of phosphorylated receptor and its CTF (Fig. 3C, middle panel), consistent with DDR1b activation and processing.

Previous reports found that DDRs elicit anti-proliferative effects on various cell types cultured on COL1 matrices^48,74–78^. However, our xenograft studies demonstrated a pro-growth effects of DDRs and COL1. Therefore, we wished to evaluate the effects of DDR1b and DDR2 in the proliferation of HT-DDR1b and HT-DDR2 cells cultured in 2D or 3D COL1 conditions. In agreement with the previous reports^48,74–78^, DDR1b or DDR2 expression was consistently associated with a significant decrease in cell proliferation in 2D (Fig. 3E,F) or 3D (Supplementary Fig. 4) COL1 conditions. This effect could not be ascribed to high levels of recombinant DDR expression in the HT1080 system because in the absence of COL1, the recombinant DDRs had no impact on *in vitro* cell proliferation (Fig. 2E,F). Taken together, the xenograft and cultured studies revealed divergent effect of DDRs and COL1 in HT1080 cell growth *in vivo* and *in vitro*.

### DDRs accelerate HT1080 tumour growth rate only with COL1

**Figure 4:**
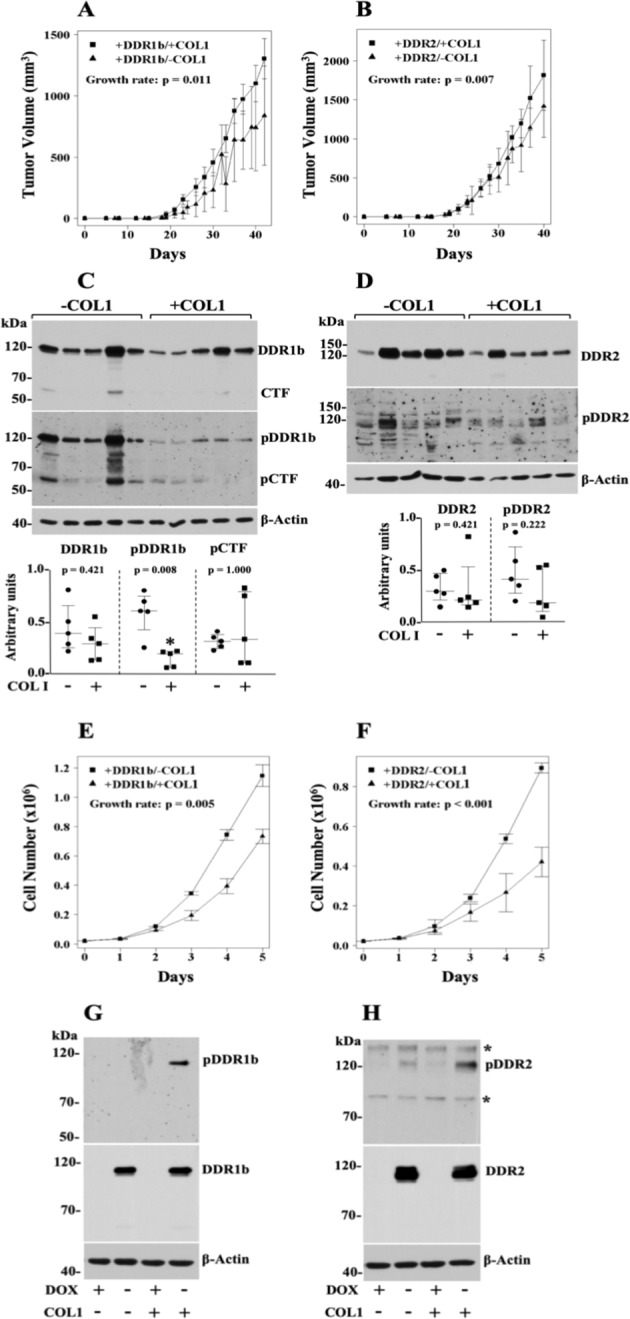
DDRs promote HT1080 tumour growth rate only when cells are co-inoculated with COL1. (**A**,**B**) Volumes of +DDR/±COL1 tumours as a function of time. HT-DDR1b (**A**) and HT-DDR2 (**B**) cells were incubated for two days without DOX to induce DDR1b or DDR2 expression. Then, the cells were harvested, and a fraction of the cells was mixed with an ice-cold solution of rat-tail COL1 (2 mg/ml, final concentration) whereas the other fraction was mixed with serum-free media. Mice fed a regular diet, were subcutaneously inoculated with 100 μl of the cell suspension (with or without COL1) containing 1 × 10^6^ cells. Number of mice for each group is provided in Supplementary Table 1. Tumours were measured every 2–3 days, and tumour volumes were calculated. Tumour growth rates were determined, as described in the Methods section. Note: The +DDR/−COL1 groups here are the+DDR groups plotted in Fig. 2A,B, and the +DDR/+COL1 groups are also plotted in Fig. 3A,B. (**C**,**D**) Expression and activation of DDRs in +DDR/±COL1 tumour extracts. Tumour extracts of +DDR1b/±COL1 (**C**) and +DDR2/±COL1 (**D**) tumours (n = 5 for each group) in RIPA buffer were resolved by reducing SDS-PAGE followed by immunoblot analyses using antibodies against phosphorylated DDR1b (Y513) (**C**, middle panel) or DDR2 (Y740) (**D**, middle panel). The blots were then stripped and reprobed with antibodies to total DDRs (upper panels), and then reprobed for β-actin as loading control (lower panels). Blots were quantified as described in the Methods section. Scatter plots show median with interquartile range where each dot represents an individual tumour. Mann-Whitney U test was performed for statistical analyses. Full-length blots are presented in Supplementary Fig. 12. (**E**,**F**) *In vitro* cell proliferation of +DDR-expressing HT1080 cells in 2D COL1 vs. plastic. HT-DDR1b (**E**) and HT-DDR2 (**F**) cells were incubated without DOX for 3 days and then 2 × 10^4^ cells/well were seeded on 24-well plates coated with or without fibrillar COL1 (100 μg/well), in triplicates, in complete media. At various time points, the cells were detached and counted with a Coulter counter. Results represent the average of three independent experiments and data plotted as described in Methods. Note: The +DDR/−COL1 groups here are the +DDR groups plotted in Fig. 2E,F, and the +DDR/+COL1 groups are also plotted in Fig. 3E,F. (**G**,**H**) Expression and activation of DDRs in HT1080 cells ±DDRs cultured for 5 days in 2D COL1 or plastic. HT-DDR1b (**G**) and HT-DDR2 (**H**) cells were incubated for three days with (+) or without (−) DOX to repress or induce DDR1b or DDR2 expression, respectively. Then, 2 × 10^4^ cells/well were seeded on 24-well plates, on uncoated wells (−) or wells coated with fibrillar COL1 (100 μg/well) (+), in triplicates. The cells were then cultured in complete media for 5 days, and then lysed in RIPA buffer. Equal protein amounts per lane (20 µg) were resolved by reducing 7.5% SDS-PAGE followed by immunoblot analyses using antibodies to phosphorylated DDR1b (Y513) (pDDR1) or DDR2 (Y740) (pDDR2). The same blots were then stripped and reprobed with antibodies against total DDRs (middle panels), and against β-actin, as loading control (lower panels). Asterisks indicate non-specific bands. Full-length blots are presented in Supplementary Fig. 12.

The studies of Fig. 3 compared the growth rate of ±DDR/+COL1 tumours (variable DDRs), which demonstrated that COL1 accelerated tumour growth only in cells expressing DDRs. We then wished to compare the growth rate of +DDR/±COL1 tumours (variable COL1), in order to evaluate the importance of COL1 on the ability of DDRs to promote s.c. tumour growth. As depicted in Fig. 4A,B, the tumour growth rate of +DDR1b/+COL1 and +DDR2/+COL1 tumours was significantly faster than that of +DDR tumours without COL1 (*p* = *0.011* for DDR1b and *p* = *0.007* for DDR2) (summarized also in Supplementary Table 1). These results further indicated that the accelerated growth rate of HT1080 tumours could not be achieved by DDRs alone and was only uncovered by the combination of DDRs and COL1.

Immunoblot analyses were performed to confirm expression and activation of DDRs in these tumours. As shown in Fig. 4C,D, the +DDRs ± COL1 tumours demonstrated expression of the recombinant DDRs in the tumour extracts, as expected. Interestingly, the +DDR1b/+COL1 tumours displayed a significant decrease in phosphorylated DDR1b (at Y513) when compared to +DDR1b/−COL1 tumours (Fig. 4C, *p* = *0.008*). Because reduced levels of DDR1b may be a consequence of receptor processing^27,63–65^, we examined the levels of total and phosphorylated CTF in these samples. These analyses did not reveal significant differences in levels of total or phosphorylated CTF between these two groups, under these conditions. The +DDR2/+COL1 tumours showed no reduction in total DDR2. However, they displayed a slight decrease in phosphorylated DDR2 (at Y740) when compared with +DDR2/−COL1 tumours, which was not statistically significant (Fig. 4D).

Next, we compared the same parameters, +DDR/±COL1, on cell proliferation under 2D COL1 conditions *in vitro*. These data demonstrated that DDR-expressing cells cultured in 2D COL1 exhibited reduced cell proliferation when compared to DDR-expressing cells growing on uncoated (plastic) wells (Fig. 4E,F). Again, the inhibitory effect of DDRs on *in vitro* cell proliferation were only manifested in the presence of COL1. To examine the status of DDR phosphorylation under those conditions, we collected cells growing in 2D COL1 or plastic at the 5-day time point. These analyses, depicted in Fig. 4G,H, showed readily detectable levels of DDR1b and DDR2 phosphorylation in COL1, consistent with the known sustained collagen-evoked DDR activation^2^. On plastic, no detectable DDR1b receptor activation was observed in HT-DDR1b cells (Fig. 4G), but background DDR2 activation was detected in HT-DDR2 cells (Fig. 4H), possibly caused by the presence of serum. Indeed, HT1080 cells do not express collagen type I, as previously shown^79,80^ and confirmed by us (data not shown). Regardless, these analyses demonstrated that slow proliferating HT1080 cells in 2D COL1 conditions were able to display detectable and sustained DDR activation even after 5 days in culture.

### COL1 has no impact on HT1080 tumour growth in the absence of recombinant DDR induction

Because COL1 matrices can positively influence tumour growth *in vivo*^81–84^, we further examined any potential effects of COL1 on s.c. HT1080 tumour growth independent of DDR induction. To this end, we compared the growth rate of HT-DDR1b and HT-DDR2 cells co-implanted with or without COL1 in mice fed a +DOX diet (DDR repression). These studies showed that, under conditions of DDR repression, COL1 had no impact on tumour growth rates in both HT-DDR1b and HT-DDR2 cells (Supplementary Fig. 5A,B, respectively). Thus, in the absence of recombinant DDR induction (+DOX), COL1 did not accelerate HT1080 tumour growth rate. Taken together, these studies uncovered a pro-tumorigenic effect of DDRs in HT1080 cells that was only manifested upon co-inoculation of cells within a scaffold of COL1. Consistently, sole induction of DDR expression (Fig. 2A,B) or presence of COL1 in the absence of DDR induction (Supplementary Fig. 5A,B) was not sufficient to accelerate tumour growth rate, indicating that the effects observed could not be attributed to (i) high levels of recombinant receptor expression or to (ii) the presence of a supportive matrix. Rather, accelerated growth rate was solely the result of specific interactions of DDRs with COL1. It is important to note that HT1080 cells also express collagen-binding integrins^85,86^. Yet, these receptors were not sufficient to enhance tumour growth when the cells (with repressed DDRs) were co-implanted with COL1. Thus, under the experimental conditions, the interactions of DDRs with COL1 were the predominant promoters of tumour growth. However, at this junction, a crosstalk between DDRs and integrins cannot be ruled out^14^. Although here we focused on the DDR1b isoform, one of the three functional full-length DDR1 receptors^4,6,87^, it will be important in future studies to address the relative contribution of the other DDR1 isoforms on tumour growth.

### DDR1b but not DDR2 is a potent suppressor of experimental lung metastases of HT1080 cells

**Figure 5:**
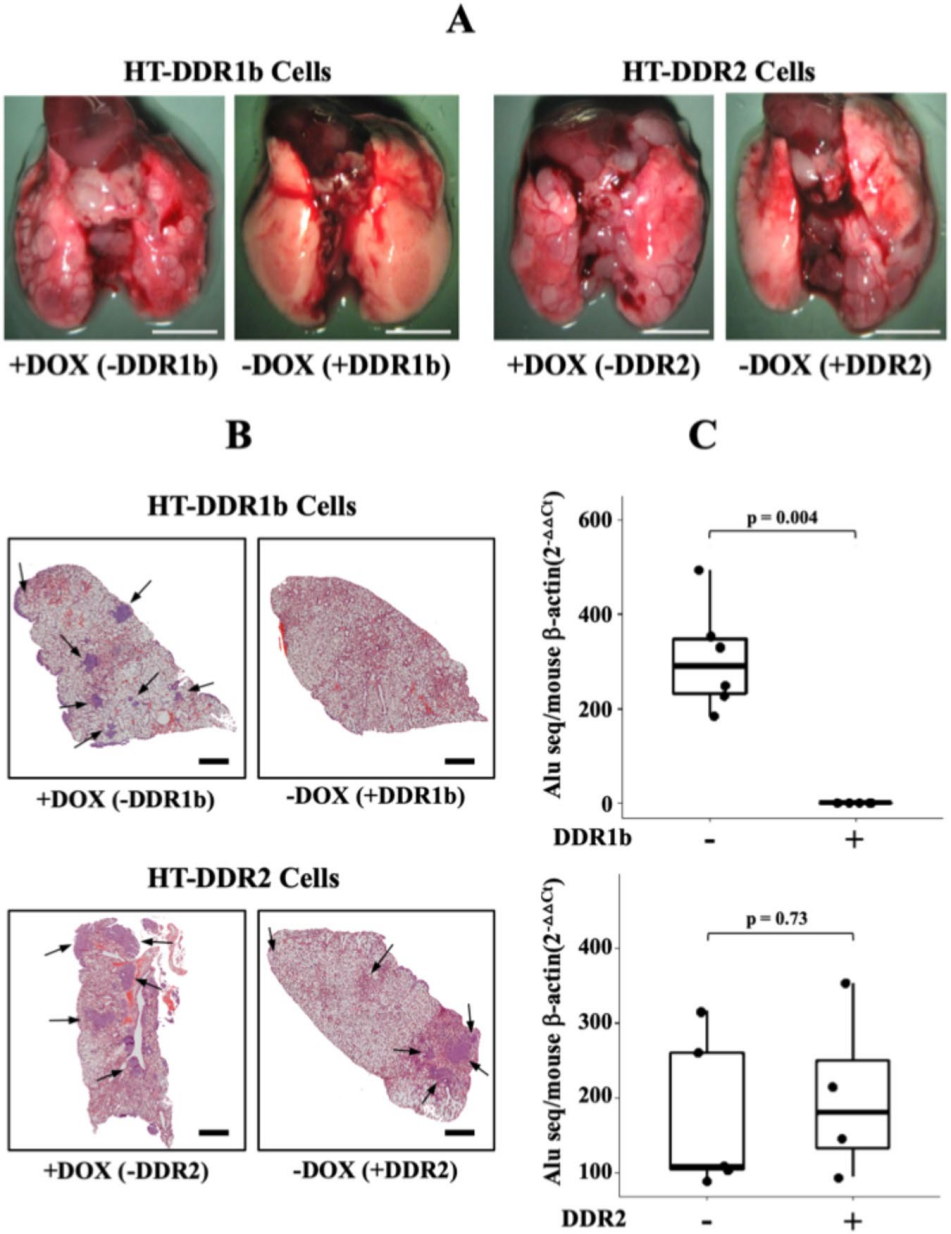
DDRs differentially regulate the lung colonizing ability of intravenously inoculated HT1080 cells. HT-DDR1b and HT-DDR2 cells were incubated for two days with or without DOX to repress or induce DDRs, respectively. Then, the cells were harvested, and 1 × 10^6^ cells/mouse were inoculated into the tail vein of mice that were fed a regular or a DOX-supplemented diet. After ~20 days, mice were euthanized, and the lungs were harvested and examined for macroscopic (**A**) and microscopic (**B**) tumour colonies, and for human DNA *Alu* sequences (**C**). (**A**) Gross anatomical photos of lungs of mice inoculated with either HT-DDR1b or HT-DDR2 cells ±DOX treatment. (**B**) H&E sections of lungs from mice inoculated with HT-DDR1b or HT-DDR2 cells ±DOX treatment. Arrows indicate tumour cell colonies. (**C**) Human *Alu* qPCR analyses of lungs (right three lobes) of mice inoculated with either HT-DDR1b or HT-DDR2 cells. −DDR1b (n = 6), +DDR1b (n = 5), −DDR2 (n = 5), and +DDR2 (n = 4). Statistical analyses were performed using Mann-Whitney U test, as described in the Methods section.

Having evaluated DDRs’ role in HT1080 s.c. tumour growth, we wished to examine the role of DDRs in metastatic dissemination, the late step of cancer progression. However, because HT1080 cells do not spontaneously metastasize from s.c. sites, we utilized the experimental lung metastasis assay, in which tumour cells are directly inoculated into the blood circulation through the tail vein. It is well established that HT1080 cells are highly metastatic to the lungs when inoculated intravenously. To this end, HT-DDR1b and HT-DDR2 cells with or without DDR expression were intravenously inoculated into mice fed a diet with or without DOX supplementation, as described in Methods. To quantitatively estimate metastatic tumour burden of human HT1080 cells within the mouse lung parenchyma we took advantage of the presence of specific *Alu* sequences in human DNA^60^. As depicted in Fig. 5A, macroscopic examination of the lungs revealed readily detectable tumour nodules in lungs of mice inoculated with HT-DDR2 cells regardless of DDR2 expression (−DOX and +DOX). Furthermore, microscopic evaluation of lung sections (Fig. 5B, bottom panels) and *Alu* qPCR analyses (Fig. 5C, bottom graph) revealed significant and similar tumour burden in both +DDR2 and −DDR2 cells. Lungs of mice inoculated with HT-DDR1b cells with repressed DDR1b (+DOX) exhibited numerous macroscopic tumour nodules in the lungs (Fig. 5A, first panel from the left), as expected from the metastatic HT1080 cell line. Surprisingly, lungs of mice inoculated with DDR1b-expressing HT-DDR1b cells (−DOX) displayed no clear evidence of macrometastases on the lung surfaces (Fig. 5A, second panel from the left) of all mice tested. Moreover, thorough microscopic analyses of lung tissue sections, and immunohistochemical staining for human DDR1 (data not shown), revealed no clear evidence of tumours cell nests within the lung parenchyma (Fig. 5B, upper panel). Consistently, these lungs showed no significant detection of human *Alu* sequences (Fig. 5C, top graph). Thus, DDR1b, but not DDR2, expression markedly reduced pulmonary colonies of HT1080 cells.

Because establishment of lung metastases requires invasion through extracellular matrices, we asked whether the differential effects observed *in vivo* could be related to differences in invasiveness. To address this question, we utilized the Matrigel invasion assay and the spheroid collagen invasion assay, two established methods to evaluate invasiveness *in vitro*. We found that neither DDR1b nor DDR2 induction altered the number of invading HT1080 cells through Matrigel-coated filters, when compared to cells with repressed DDR expression (Supplementary Fig. 6A,B). Next, we evaluated invasive activity using the spheroid invasion assay, with COL1 as a matrix barrier^58^. To this end, HT-DDR1b and HT-DDR2 cells were cultured in the presence of DOX to repress DDR expression, before and after seeding into the ULA plates. Under these conditions, both HT-DDR1b and HT-DDR2 cells generated tightly bound cell spheroids (Supplementary Fig. 6C). COL1 was then added to each well, and the cells were then allowed to invade the 3D matrix for two days, in the presence or absence of DOX. These studies showed that, regardless of the expression of DDR1b or DDR2, the area invaded by the HT1080 cells was comparable in all conditions (Supplementary Fig. 6D). Thus, DDR expression did not alter HT1080 cell invasiveness through two distinct matrices. These results suggest that tumour cell invasive capacity was not likely the limiting step for the reduced ability of DDR1b-expressing HT1080 cells to generate lung metastatic colonies. Regardless, these studies further demonstrate that DDRs differently regulate two major processes, namely tumour growth and experimental metastases, in the HT1080 model of malignant cell behaviour.

### Signalling pathways in ±DDR/+COL1 HT1080 tumours

To begin elucidating the signalling pathways underlying the ability of DDRs to promote tumour growth in the presence of COL1, we focused on three major signalling networks, namely the MAPK, PI3K/AKT, and the Hippo pathways, known to be important for cell proliferation. These analyses were conducted between ±DDR tumours generated by cells co-inoculated with COL1 because only under those conditions DDRs stimulated tumour growth. Evidence suggest that DDRs can influence cell behaviour, including cell proliferation, through the activation the MAPK and the PI3/AKT signalling pathways^1,5,29,66,74,88–91^. Therefore, we evaluated the levels of total and phosphorylated ERK1/2 and AKT in ±DDR/+COL1 tumours by immunoblot analyses. These analyses revealed no differences in levels of pERK1/2 (T202/Y204) in both +DDR1b/+COL1 and +DDR2/+COL1 tumours when compared to those with repressed DDRs (Supplementary Fig. 7A,B). In contrast, we found that only +DDR2/+COL1 tumours showed significantly higher levels of pAKT-S473 (Supplementary Fig. 7C,D), compared to −DDR2/+COL1 tumours, suggesting that AKT-mediated signalling plays a role in the stimulation of tumour growth in the +DDR2/+COL1 xenografts.

**Figure 6:**
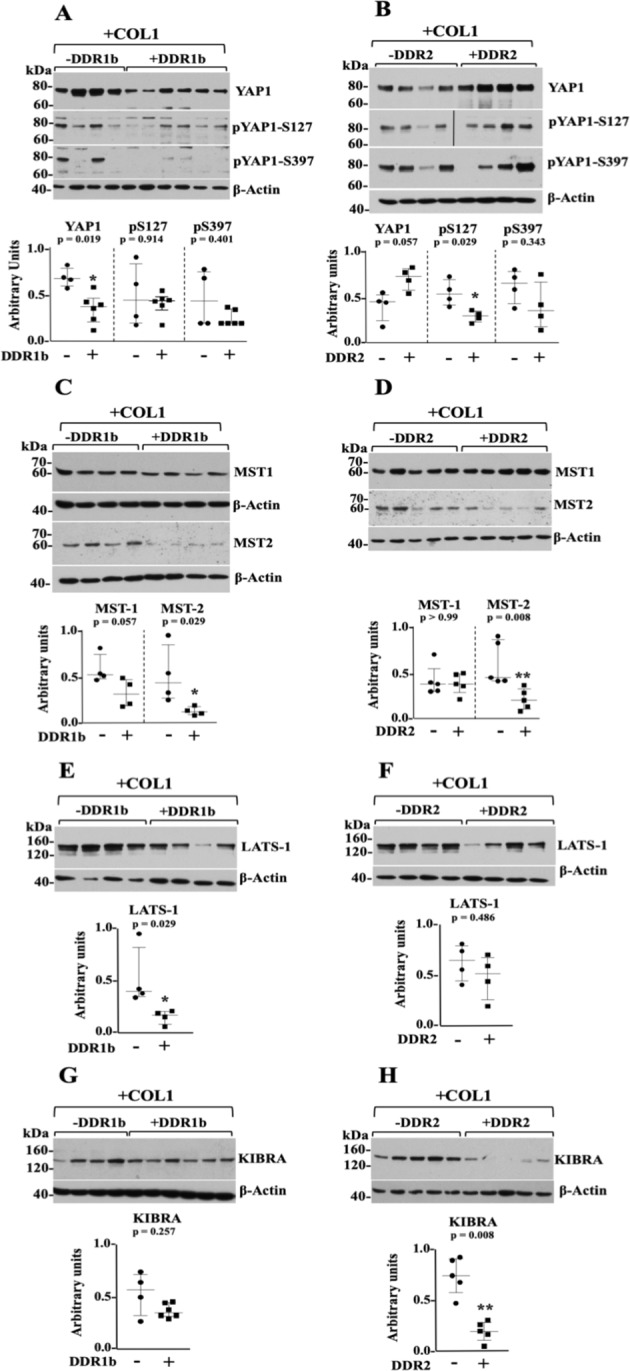
Impact of DDRs on Hippo pathway components in HT1080 ± DDR/+COL1 tumours. (**A–H**) Immunoblots of Hippo core components. Extracts of HT-DDR1b tumours (**A**,**G**) −DDR1b and +DDR1b, n = 4 and n = 6 each, respectively; (**C**,**E**) ±DDR1b, n = 4 each) and HT-DDR2 tumours (**B**,**F**) ±DDR2, n = 4 each; (**D**,**H**) ±DDR2, n = 5 each) that were co-inoculated with COL1, and harvested at time of sacrifice, were resolved by reducing SDS-PAGE followed by immunoblot analyses using antibodies to the indicated Hippo pathway components: YAP-1, pYAP1-S127, and pYAP1-S397 (**A**,**B**); MST1 and MST2 (**C**,**D**); LATS1 (**E**,**F**); and KIBRA (**G**,**H**). Blots were probed with the indicated antibodies and quantified as described in the Methods section. Note: in panels **A** and **B**, the blots for pYAP1-S127 and pYAP1-S397 were each run in separate blots with the same tumour extract samples. Each blot was then stripped and reprobed for total YAP1. Then, the relative levels of phosphorylated to total YAP1 levels were each quantified with their respective blot. However, in the panels only one total YAP1 blot for DDR1b or DDR2 is shown. In panel **B**, the +DDR2 blot for pYAP1-S127 comprises samples that were run in the same blot. However, an empty lane in the middle was cropped and it is now indicated by a vertical black line. Scatter plots show median with interquartile range where each dot represents an individual tumour. Mann-Whitney U test was performed for statistical analyses. Full-length blots are presented in Supplementary Fig. 13.

Next, we focused on the Hippo pathway because this signalling pathway is regulated in part by mechanical signals initiated by the biophysical properties of ECM components, including collagens^53,54^. Moreover, DDR1 activation, in two mouse fibroblastic cell lines, was shown to be regulated by collagen stiffness, suggesting a role for DDR1 in mechanosensing^92^, and as a potential regulator of the Hippo pathway. The Hippo signal transduction pathway is also major player in the progression of many cancer types^93–96^, including sarcomas^97–100^. To this end, extracts from 4–6 representative tumours were processed for immunoblot analyses to examine the total levels and phosphorylation status of Yes-associated protein (YAP; here isoform YAP1), a major effector of the Hippo pathway^54^. Phosphorylation of human YAP1 at S127 creates a binding site for 14–3–3 proteins, leading to retention of YAP1 in the cytoplasm, whereas phosphorylation at S397 leads to β-TrCP-mediated proteasomal degradation of YAP^101^. Increased levels and/or reduced YAP phosphorylation have been associated with pro-oncogenic effects of YAP in various cancer types, including sarcomas^97,102,103^. Here we found that +DDR1b tumours exhibited significantly lower levels of total YAP1 (~75 kDa) when compared to -DDR1b tumours (Fig. 6A, upper panel, *p* = *0.019*). In contrast, +DDR2 tumours displayed higher levels of total YAP1 when compared to -DDR2 tumours (Fig. 6B, upper panel, *p* = *0.057*). Quantification of pYAP1 at S127 and S397 revealed that levels of pYAP1-S397 were not altered in both +DDR1b and +DDR2 tumours, when compared to tumours with no DDR induction (Fig. 6A,B, third panels from the top, *p* = *0.401* and *0.343*, respectively). However, +DDR2 tumours showed a significantly lower phosphorylation of YAP1 at the S127 site (Fig. 6B, second panel from the top, *p* = *0.029*), whereas +DDR1b tumours showed no difference (Fig. 6A, second panel from the top, *p* = *0.914*).

The Hippo pathway also include a set of kinases, namely the Ser/Thr kinases MST1/2 (mammalian Ser/Thr sterile 20 (Ste20)-like kinases 1/2) and the LATS (Large Tumour Suppressor) kinases^54,104^. The MST1/2 kinases phosphorylate and activate the LATS kinases, which in turn phosphorylate YAP and TAZ resulting in the inactivation of their pro-oncogenic effects^54,104,105^. A frequent loss of these kinases, at multiple levels of regulation, has been shown to be a characteristic of human tumours, including sarcomas^97,99,103,106^. Therefore, we examined the levels MST1/2 and LATS1 kinases in ±DDR tumours +COL1. We found that MST2 (~60 kDa) (Fig. 6C, third panel from the top, *p* = *0.029*), and LATS1 (~140–150 kDa) (Fig. 6E, *p* = *0.029*) were both significantly downregulated in +DDR1b tumours when compared to −DDR1 tumours. MST1 levels in +DDR1b tumours were also decreased. However, this decrease was marginally significant (Fig. 6C, upper panel, *p* = *0.057*). In contrast, +DDR2 tumours showed significant downregulation of MST2 (Fig. 6D, *p* = *0.008*) and no differences in MST1 (Fig. 6D, *p* > *0.99*) or LATS 1 (Fig. 6F, *p* = *0.486*) levels when compared to −DDR2 tumours. Thus, both +DDR1b and +DDR2 tumours in COL1 share a common and significant downregulation of MST2.

Next, we examined the expression of Kidney and Brain Protein (KIBRA), a WW domain-containing protein that is an upstream regulator of the Hippo core kinases that contributes to LATS1/2-mediated YAP phosphorylation^107–109^. A previous study showed that in the human HEK293 epithelial cell line, unstimulated DDR1 can form a complex with KIBRA, whereas collagen-induced receptor activation leads to dissociation of the DDR1/KIBRA complex^25^. Here, we found that +DDR2 tumours showed a consistent and significant downregulation of KIBRA (~140 kDa) when compared to -DDR2 tumours (Fig. 6H, *p* = *0.008*). In contrast, the level of KIBRA in +DDR1b tumours was only slightly decreased when compared to −DDR1b tumours (Fig. 6G, *p* = *0.257*). Thus, in this model system, +DDR2, but not +DDR1b, tumours show a strong downregulation of KIBRA.

**Figure 7:**
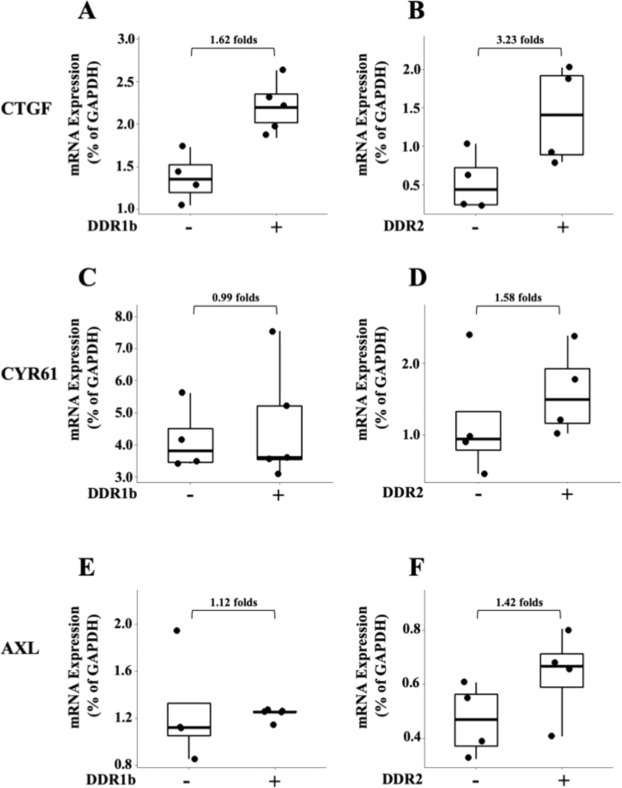
Impact of DDRs on YAP1 transcriptional targets in HT1080 ± DDR/+COL1 tumours. Total RNA was isolated from s.c. ±DDR1b (**A**,**C**,**E**) and ±DDR2 (**B**,**D**,**F**) tumours (n = 4 and 5 for −DDR1b and +DDR1b tumours, respectively, and n = 4 for both –DDR2 and +DDR2 tumours) and qPCR analyses for human CTGF (**A**,**B**), CYR61 (**C**,**D**) and AXL (**E**,**F**) were conducted as described in the Methods section. Folds of mRNA expression between ±DDR tumours are indicated.

Inactivation of the Hippo pathway leads to YAP translocation to the nucleus and subsequently YAP-mediated transcriptional activation of genes involved in tumorigenesis and metastases^50^. Analyses of YAP1 subcellular distribution in tissue sections of +DDR/+COL1 tumours by IHC, however, failed to reveal a clear-cut increase in nuclear over cytoplasmic YAP1 localization when compared to −DDR/+COL1 (not shown). Possibly, the fact that HT1080 xenografts are, in general, highly proliferative tumours, makes it challenging to detect a clear differential subcellular distribution of rapidly shuttling YAP1 by IHC^110^. Next, we examined the expression of three known YAP targets, namely connective tissue growth factor (CTGF), cysteine-rich angiogenic inducer 61 (CYR61) and Axl receptor tyrosine kinase (AXL)^111^. We found that +DDR1b/+COL1 and +DDR2/+COL1 tumours displayed a 1.62- and 3.23-fold increase in CTGF mRNA levels, respectively, when compared to −DDR1b or −DDR2 tumours in COL1, (Fig. 7A,B). +DDR2/+COL1 tumours also showed higher levels of CYR61 and AXL (1.58 and 1.42 folds, respectively) than −DDR2/+COL1 tumours (Fig. 7D,F). In contrast, CYR61 and AXL levels were not increased in +DDR1/+COL1 tumours when compared to −DDR1/+COL1 tumours (Fig. 7C,E). Thus. both DDR1b and DDR2 increased the levels of CTGF, a key matricellular protein in cancer progression and fibrosis^112^. Importantly, CTGF has been implicated in the malignant phenotype of HT1080 cells^98,113,114^.

Next, we wished to examine the profile of Hippo pathway components as a function of DDR expression independent of COL1, namely in ±DDR/−COL1 tumours, which showed no differences in tumour growth rate, size or latency as a function of DDR expression (Fig. 2A,B, and Supplementary Table 1). Immunoblot analyses of the ±DDR/−COL1 tumours revealed that neither DDR1b nor DDR2 expression altered the levels of either YAP1 and its phosphorylated forms (Supplementary Fig. 8A,B), MST1 and MST2 (Supplementary Fig. 8C,D), LATS1 (Supplementary Fig. 8E,F) or KIBRA (Supplementary Fig. 8G,H), when compared to tumours with repressed DDRs (+DOX). These results suggest that the significant downregulation of MST1/2 and LATS1 in +DDR1b/+COL1 tumours and MST2 and KIBRA in +DDR2/+COL1 tumours are specific Hippo pathway alterations elicited by DDRs in conjunction with COL1.

## Discussion

Tumour cell-collagen interactions are known to play pivotal roles in cancer progression^72,73,115–119^. This notion was established, in part, in multiple studies with xenograft models of cancer in which the composition and biophysical properties of the collagenous matrix were altered by modulating the synthesis, cross-linking, cleavage, and stiffness of collagen^119–127^. Also, a number of studies utilized collagen I gels to inoculate tumour cells *in vivo*^81–84^, demonstrating a tumour-promoting effect of collagen. Yet, evidence indicate that collagen matrices can elicit divergent effects on tumour cells by either promoting or suppressing malignant activity^128^. For instance, collagen matrices can restrict tumour cell proliferation^129–136^. Consistent with this view^128^, we showed that ectopic expression of DDR1b or DDR2 in HT1080 cells elicited a potent growth inhibitory effect only when the cells were cultured on 2D or 3D COL1 matrices, in agreement with previous studies in melanoma^48^, breast cancer^76,78^, and lung cancer cells^74,75^. The mechanisms for the anti-proliferative effects of DDRs and COL1 *in vitro* are still poorly understood, however previous studies have shown that this process may include induction of cell cycle arrest^48,75^ or apoptosis^77,78^. While *in vitro* DDRs and COL1 reduced cell proliferation, this effect was not recapitulated in tumour xenografts, in which DDR-expressing HT1080 cells co-implanted within a COL1 scaffold displayed accelerated tumour growth rate. These observations suggest that DDRs are part of the oncogenic programs activated by cancer cells when confronted with a dense ECM. How do DDRs and COL1 change from being repressor to become stimulators of tumour cell growth? Because DDRs are collagen receptors, this may be caused in part by changes in the structural and mechanical properties of the collagen matrix^29,49,77^. For instance, rat collagen type I isolated from old rats, as opposed to adult collagen I, was shown to override DDR-dependent inhibition of cell growth in 3D collagen^49,77^, indicating that the structure of the collagen matrix may alter DDR response. Conversely, alterations in receptor expression, structure, or integrity can also turn DDRs into growth stimulators. In the case of DDR2, specific gene mutations reversed collagen-dependent inhibition of cell growth in human embryonic kidney HEK-293 cells and human lung cancer A549 cells^74,75^ and enhanced colony formation in mouse fibroblast NIH3T3 cells^137^, consistent with a suppressor activity of DDR2. Interestingly, some of these mutations also reduced collagen-evoked DDR2 phosphorylation^74,75^, suggesting that receptor activation can elicit anti-growth signals. The suppressive effects of DDR1 on cell growth, on the other hand, may be overridden by proteolytic cleavage of the receptor. Indeed, DDR1, as opposed to DDR2, is uniquely sensitive to proteolytic cleavage^27,63–65^. Consistently, MT1-MMP-dependent DDR1 cleavage has been recently shown to restore cell growth on collagen type I^49,76,78^. Interestingly, in the context of collagen modifications and receptor cleavage, recovery of cell proliferation in DDR1-expressing cells in collagen type I matrices was associated with decreased receptor phosphorylation^77,78^. In agreement with these observations, we found that +DDR1b/+COL1 tumours, which grew faster than +DDR1b/−COL1 tumours, showed reduced levels of phosphorylated DDR1b when compared +DDR1b/−COL1 tumours (Fig. 4C). However, reduced DDR1b phosphorylation did not correlate with increased levels of cleaved receptor. Thus, other mechanisms are likely to play a role in the attenuation of DDR1b activation (phosphorylation). These may include receptor endocytosis and/or phosphatase activity, just to mention a few. Regardless, although the reduced activation of DDR1b in the +DDR1b/+COL1 tumours could be interpreted to suggest that the extent of DDR1b phosphorylation is inversely associated with tumour growth rate, it is worth pointing out that receptor activation in these tumours is still detectable at the time of tumour harvesting. Conversely, under *in vitro* 2D COL1 conditions, which significantly inhibits cell proliferation, receptor activation was clearly detectable even after 5 days in culture (Fig. 4G–H). However, *in vitro*, a comparable control for the extent of DDR activation was not feasible, because the recombinant DDRs displayed no detectable activation in cells cultured on plastic (Fig. 4G–H), precluding a fair comparison of DDR phosphorylation and cell proliferation under those conditions. Regardless, based on previous reports^74,75,77,78^ and taking into account that our DDR phosphorylation data focused on a single phosphorylation site for each receptor, the relationship between the extent of collagen-evoked DDR overall phosphorylation and cell growth appears to be complex; depending among other factors on the cellular context, the DDR type, the dynamics of receptor phosphorylation at distinct sites, and the environmental conditions, just to mention a few. More studies are warranted to elucidate the relationship between DDR phosphorylation and the growth of cancer cells within collagen matrices.

Here we showed that the ability of COL1 scaffolds to promote tumour growth in HT1080 xenografts was only manifested in the context of ectopic DDR1b or DDR2 expression because co-implantation of COL1 with HT1080 cells in which either DDR1b or DDR2 expression was repressed (+DOX) had no impact on tumour growth (Fig. 2A,B). Thus, presence of high concentration of COL1 (2 mg/ml) at the site of tumour implantation was not sufficient to confer a growth advantage without a predisposing threshold of DDR expression. This is consistent with the notion that, in the absence of a relevant protein profile, collagen networks are not sufficient to activate pro-malignant programs in tumour cells^138^. Conversely, our data demonstrate that, in the model used, expression and activation of tumour-derived DDRs were not sufficient to enhance tumorigenicity without the support of an exogenously supplied COL1 scaffold. Likewise, presence of COL1 without DDR expression had no effect on tumour growth, demonstrating the specificity of the COL1/DDR effect. Thus, these results uncovered a unique crosstalk between DDRs and collagen in the promotion of HT1080 tumour growth that could not be recapitulated by either DDRs or COL1 alone. Having said that, it is important to note that DDRs display different selectivity toward collagen ligands^1,2^. Thus, it will be interesting to evaluate the effects of other matrices such as Matrigel, which contains COL4, a ligand for DDR1 but not DDR2, to further address the specificity of DDR-ECM interactions in regulation of tumour growth. Moreover, the fact that the biomechanical and biophysical properties of COL1 scaffolds can be altered^139,140^, it will be important to address DDR function *in vivo* under various matrix conditions that may best resemble those found within the tumour microenvironment. Indeed, the fact that DDRs are matrix receptors makes them uniquely susceptible to and dependent of microenvironmental factors, which if limited, receptor function is significantly altered. This is exemplified by the dichotomy between the anti- and pro-proliferative effects of DDRs *in vitro* vs. *in vivo* conditions.

Previous studies have addressed the roles of DDRs in experimental models of metastases, which showed a consistent pro-metastatic role for DDR2. In contrast, conflicting results have been reported on the ability of DDR1 to support metastatic dissemination (reviewed in^5,87^). For instance, genetic deletion of DDR1 increased pulmonary metastases in the MMTV-PyMT model of mammary carcinoma in mice^141^. In contrast, ectopic expression of DDR1b in the human T24 bladder cancer cell line elicited a pro-metastatic effect in both spontaneous and lung experimental metastases assays, which was ascribed to an effect on collagen III-mediated regulation of DDR1b within the lung parenchyma^45^. Our studies in the HT1080 cell system showed that ectopic expression of DDR1b, but no DDR2, markedly inhibited formation of pulmonary metastases upon intravenous inoculation. Thus, in this model system, DDR1b functions as a potent suppressor of lung colonization. Interestingly, *in vitro* invasiveness, a surrogate method for evaluating invasive ability of tumour cells showed no significant effects of DDRs in this process. Although more studies are required to determine the mechanism(s) by which DDR1b expression inhibits lung colonization in HT1080 cells, these results suggest that DDR1b may suppress formation of experimental lung metastases by disrupting the ability of HT1080 tumour cells to survive in the circulation, arrest in or extravasate through the lung capillaries, and/or proliferate within the lung parenchyma. Interestingly, despite that fibrillar collagens are part of the lung microenvironment, this ECM does not appear to be critical for supporting a lung colonizing phenotype in circulating DDR1b-expressing HT1080 cells, as reported in a bladder cancer cell line^45^. On the contrary, the data presented here suggest that DDR1b-collagen interactions within the lung parenchyma may activate a signalling pathway in HT1080 cells, which eventually inhibits the growth of disseminated cells. In this regard, it will be interesting to examine whether DDR1b expression in HT1080 cells fails to reactivate lung-residing tumour cells from dormancy, as reported in other model^34,142^. Regardless, the current data are consistent with a complex role for DDR1b in metastatic dissemination, which may be cell type- and model-dependent. Thus, the concept of DDR1 as an anti-metastatic target remains to be carefully evaluated.

The underlying signalling pathways activated by DDRs and COL1 to support HT1080 tumour growth rate was examined here by focusing on the MAPK, the PI3K/AKT and the Hippo pathways. Interestingly, despite the significant differences in growth rates, our analyses showed no significant differences in pERK1/2 status as a function of DDR expression in +COL1 tumours, suggesting that the MAPK pathway may not be implicated in this model. However, phosphorylation of AKT (at S473) was significantly higher only in +DDR2/+COL1 tumours, consistent with the role of AKT in supporting cell proliferation in cancer. The Hippo pathway has become a central signalling network in neoplastic processes driven by tumour-cell matrix interactions^143^. Therefore, it was reasonable to examine the status of the Hippo pathway in the HT1080 DDR/COL1 tumours, due to their accelerated growth rate. Focusing on a limited set of Hippo core components, we found that both +DDR/+COL1 tumours displayed a significant downregulation of MST2, while +DDR1b tumours also showed reduced levels of LATS1 and MST1. This effect was not observed in DDR repressed COL1 tumours, which indicated that this was an effect specifically mediated by interactions between DDRs and COL1. Loss of MST and/or LATS kinases, by either genomics deletions, epigenetic silencing or posttranslational regulation, is a characteristic of many types of sarcomas^97,99,100,103,106,144–146^, which are also detected in sarcoma-derived cell lines, including in HT1080 cells^99^. Functionally, the MST/LATS kinases are considered to be tumour suppressors, and consistently genetic deletion of these kinases in mice increases tumorigenicity and the development of sarcomas^145,147–149^. Hence, the reduced expression of Hippo core kinases in the +DDR/+COL1 HT1080 tumours may underlie the ability of these tumours to display accelerated growth. Consistent with the tumour suppressive role of the Hippo pathway, we found that KIBRA, an upstream regulator of the Hippo pathway, and also considered a tumour suppressor^150–153^, was significantly downregulated in +DDR2/+COL1 but not in +DDR1b/+COL1 tumours. This suggests that KIBRA may be a major target of DDR2 signalling in response to collagen *in vivo*.

A major function of the Hippo core kinases is to promote YAP1 phosphorylation to regulate its activity. Here we found a differential response in terms of YAP regulation by DDR1b and DDR2, which was not always associated with the observed reduction in Hippo core kinases. The decoupling between MST/LATS kinases and YAP1 regulation observed here is consistent with evidence showing that deregulation of these kinases does not necessarily alter YAP activity (summarized in^154^). Indeed, MST/LATS kinases can regulate downstream effector other than YAP/TAZ^104,106,145,154,155^. Conversely, YAP activity is also regulated by LATS-independent mechanisms^156–158^. These new evidence underscores the complexity of analysing YAP phosphorylation/dephosphorylation dynamics and subcellular distribution in tumour samples^110,158^, and may provide a partial explanation for the discrepancies in YAP status and kinase expression observed in the DDR-expressing tumours. Indeed, it is known that the state of the Hippo pathway (in either on or off status) is not static but rather fluid, and it is not always directly correlated with the expected changes in each individual core component of the pathway^93,159^. This complex regulation may partly explain the inconsistencies observed here. Of note, although our data showed differences between DDR1b- and DDR2-expressing tumours in YAP1 regulation and other signalling molecules, these may not necessarily reflect differences between DDRs. Indeed, clonal variations (between HT-DDR1b and HT-DDR2 cells, see Methods) cannot be rule out as a potential reason for the observed differences between DDR1b and DDR2. Another consideration when interpreting signalling data is the fact that the analyses conducted here were performed with tumours samples collected at time of sacrifice. These advanced tumours, albeit derived from an established tumour cell line, likely contain a complex landscape of growing, apoptotic and necrotic areas, each possibly associated with a distinct profile of signalling networks. Thus, it is possible that the signalling pathway alterations observed in the +DDR/+COL1 tumours are not a direct downstream effect of DDRs, but rather a consequence of the multiple oncogenic processes activated during tumour development, which may crosstalk with DDRs to regulate distinct signalling pathways. Analyses of tumours at earlier stages will help to address the dynamics of DDR/COL1 signalling in the course of tumour growth. Regardless, being aware of the limitations of the experimental system, as discussed above, it is tempting to speculate that the differences and similarities in the status of Hippo core components observed here may reflect common and divergent effects of the two DDR types in response to collagen activation. Nonetheless, both receptors appear to inactivate the pathway through a common target (i.e. downregulation of MST2) to promote fibrosarcoma tumour growth within a collagen-rich environment. Finally, although the findings reported here need to be tested in additional models of cancer, the pro-growth effect of the DDR/COL1 axis has been recently validated in our laboratory with human pancreatic MiaPaCa-2 cells engineered to express DDR1b or DDR2 (manuscript in preparation). Thus, at least in two distinct tumour cell systems, DDRs and COL1 interact to promote tumour growth. Because both fibrosarcomas and pancreatic cancers are known to thrive within a collagen-rich environment^51,160^, our results further suggest that DDRs may play a critical role in the growth of cancers that are known to deposit an extracellular matrix rich in collagens.

## Supplementary information

Supplementary Information.
