# Supplementary material for: Discoidin Domain Receptors, DDR1b and DDR2, Promote Tumour Growth within Collagen but DDR1b Suppresses Experimental Lung Metastasis in HT1080 Xenografts
Source: Sci Rep. 2020 Feb 11;10:2309. doi: 10.1038/s41598-020-59028-w (PMC7012844; doi:10.1038/s41598-020-59028-w)
Supplement: Supplementary file 1 — Supplementary Information. [file 41598_2020_59028_MOESM1_ESM.pdf]

Supplementary Information*

Discoidin Domain Receptors, DDR1b and DDR2, Promote Tumour Growth within Collagen but DDR1b Suppresses Experimental Lung Metastasis in HT1080 Xenografts

Benjamin Wasinski[1,#], Anjum Sohail[1,#], R. Daniel Bonfil[1,2*], Seongho Kim[3], Allen Saliganan[2], Lisa Polin[3], Mohamad Bouhamdan[1], Hyeong-Reh C. Kim[1,3], Marco Prunotto[5,6,&], and Rafael Fridman[1,3,&,+]

From the Department of Pathology[1], Urololgy[2], and Oncology[3], Wayne State University School of Medicine and Karmanos Cancer Institute[1,2,3], Detroit, MI 48201, USA, Hoffmann-La Roche[5], Basel, Switzerland and School of Pharmaceutical Sciences[6], Geneva, Switzerland.

[*]Current address: Department of Pathology, College of Medical Sciences, Nova Southeastern University, Fort Lauderdale, FL 33328-2018, USA

[#] These authors contributed equally to this work.

[&]Co-senior authors

[+]Corresponding author: rfridman@med.wayne.edu

*Includes 18 Suppl. Figs. and 3 Tables.

Supplementary Figure 1

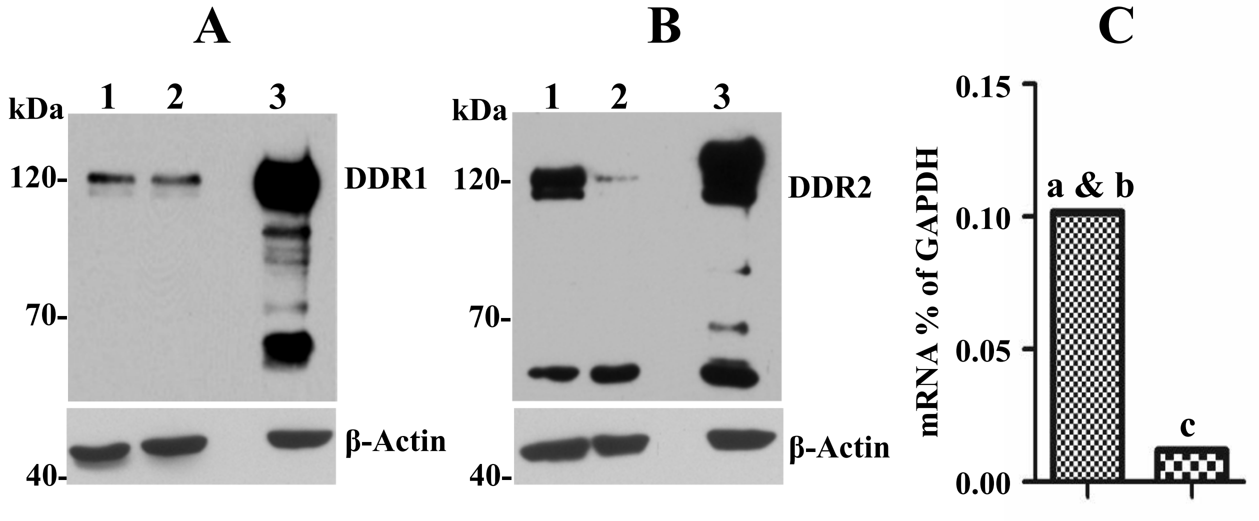

Suppl. Fig. 1. Endogenous DDR expression in HT1080 cells. DDR1 (**A**,**C**) and DDR2 (**B**) endogenous expression. (**A**,**B**) HT-DDR1b (lanes 1) and HT-DDR2 (lanes 2) cells were cultured with DOX and lysed in RIPA buffer. Lysates were then resolved by reducing 7.5% SDS-PAGE followed by immunoblot analyses using antibodies to DDR1 (Ab D1G6) (**A**), or DDR2 (Ab 12133) (**B**). The blots were then stripped and reprobed with antibodies against β-actin as loading control (lower panels). The blots were developed with SuperSignal West Femto substrate and an exposure time of ~1-2 min, to increase sensitivity in order to detect endogenous DDRs in HT1080 cells. A lysate of human pancreatic cancer CFPAC-1 cell (lane 3 in **A**) and human breast cancer BT549 cells (lane 3 in **B**) were run as positive controls for endogenous DDR1 and DDR2, respectively. Full-length blots are presented in Suppl. Figure 14. (**C**) qPCR of DDR1 isoforms (a, b, c, d, and e) were conducted with RNA isolated from HT-DDR1b + DOX and specific primers (Suppl. Table 3).

Supplementary Figure 2

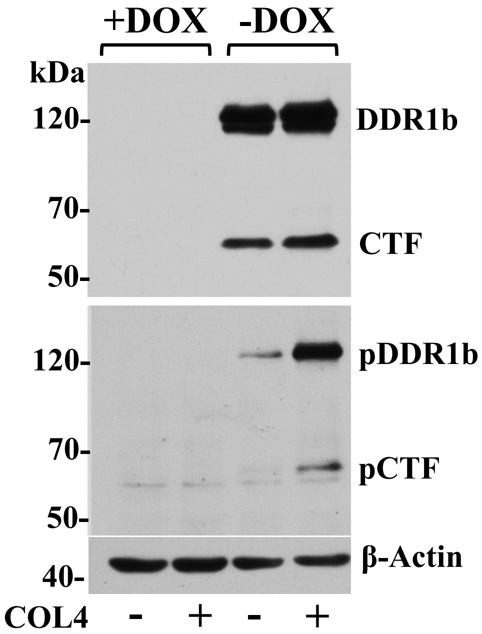

Suppl. Fig. 2. COL4 stimulation of DDR1b activation. HT-DDR1b cells were cultured with or without DOX. After three days, the cells were seeded into 60-mm dishes, and allowed to attach overnight in complete media. The next day, the cells were washed with PBS and incubated in serum-free media supplemented with or without 20 μg/ml of COL4 for 2 h at 37 °C. At the end of the incubation period, the cells were lysed in RIPA buffer and equal protein amounts per lane (30 μg) were resolved by reducing SDS-PAGE followed by immunoblot analyses using antibodies to phosphorylated DDR1b (Tyr513) (middle panel). The blots were then stripped and reprobed with antibodies against total DDR1 (upper panel), and against β-actin as loading control (lower panel). Control (Ctrl.): cells which were incubated with or without DOX but not stimulated with COL4. CTF, C-terminal fragment of DDR1b. Full-length blots are presented in Suppl. Figure 15.

Supplementary Figure 3

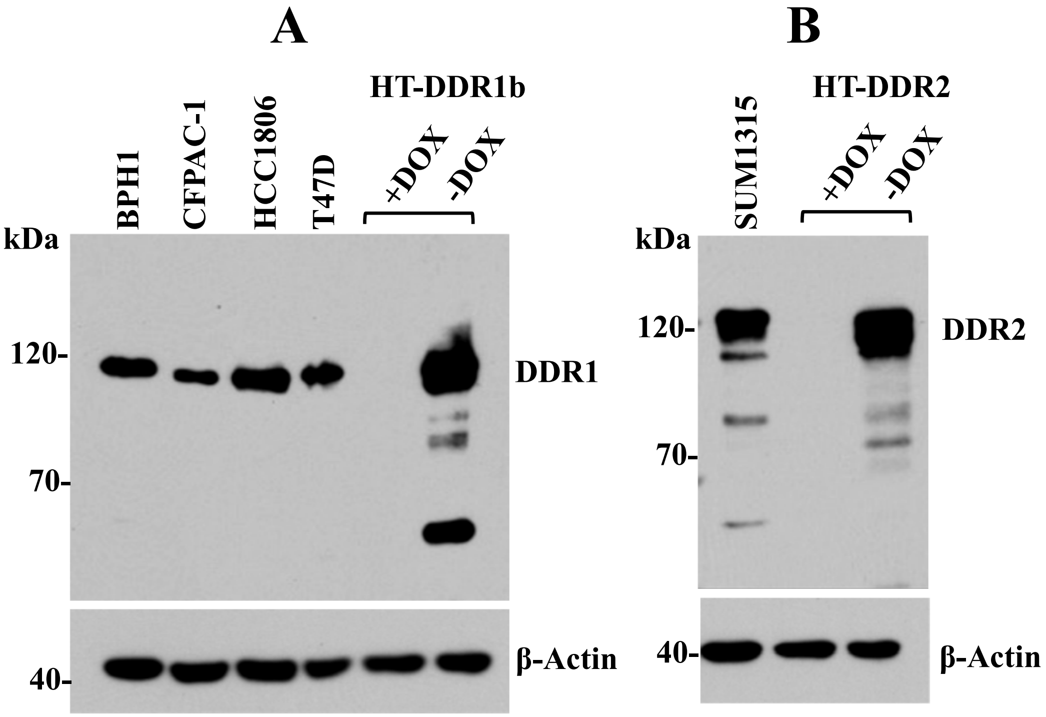

Suppl. Fig. 3. Recombinant DDR expression in HT-DDR1b and HT-DDR2 cells vs. endogenous DDR expression in human cancer cell lines. DDR1 (**A**) and DDR2 (**B**) expression was examined in HT-DDR1b and HT-DDR2 cells treated with or without DOX for three days in complete media. Cancer cell lines were cultured in complete media as described below. Cell lysates in RIPA buffer were resolved by reducing 7.5% SDS-PAGE followed by immunoblot analyses using antibodies to DDR1 (Ab D1G6) (**A**) or DDR2 (Ab 12133) (**B**). The blots were then stripped and reprobed with antibodies against β-actin as loading control (lower panels). Note that the blots were developed with a 30%/70% mixture of Femto and SuperSignal West Pico Plus substrate, respectively, and an exposure time of 15 sec. Thus, under these conditions, the endogenous DDRs in HT-DDR1b and HT-DDR2 cells are not detectable. Full-length blots are presented in Suppl. Figure16.

Cancer cell lines source and media: Human breast cancer HCC1806, BT549, and T47D cell lines were obtained from ATCC. Human breast cancer SUM1315 cells were obtained from the Karmanos Cancer Institute (Detroit, MI). Human pancreatic CFPAC-1 cells were a gift from Dr. H. Crawford lab (University of Michigan). Human benign prostate BPHI1 cells were a gift from Dr. Sheng (Wayne State University). HCC1806, BT549, T47D and BPH-1 cells were culture in RPMI-1640 with 25mM HEPES buffer (Gibco, Waltham, MA) supplemented with 8% FBS (Gibco), 1% l-Glutamine and 1% Streptomycin /Penicillin antibiotics. CFPAC-1 cells were cultured in IMDM (Gibco) supplemented with 8% FBS, 1% Streptomycin /Penicillin antibiotics. SUM1315 cells were cultured in Ham's F-12 (Gibco) with 5% FBS supplemented with insulin (5 µg/ml) (Sigma) and EGF (10 ng/ml) (Gibco). All cancer cells were collected for analyses at steady state growing conditions, in complete media.

Supplementary Figure 4

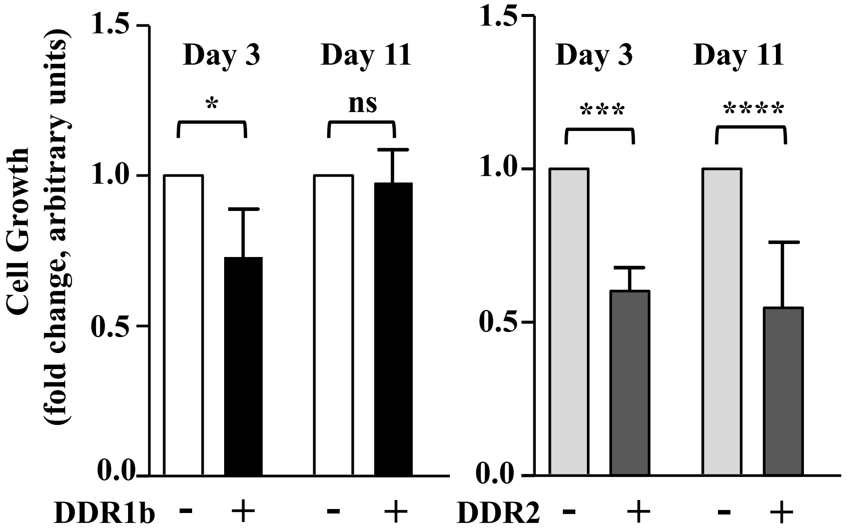

Suppl. Fig. 4. Effects of DDRs on HT1080 cell proliferation in 3D-COL1. HT-DDR1b and HT-DDR2 cells were incubated with or without DOX for three days and then they were mixed with a neutralized solution of COL1 (2 mg/ml, final concentration) in the presence or absence of DOX, in complete media. Eight replicates of the 40 µl cell-COL1 mixture were then added to a 96-well plate to a final density of 1 x 10^3 cells/well. The plates were then incubated at 37° C, 5% CO_2 to allow COL1 gelling. Next, 100 µl of complete medium with or without DOX were added to each well. Cells were then incubated at 37° C, 5% CO_2 for 3 or 11 days with a media change every 2-3 days. Cell growth was determined using the XTT assay, as described by the manufacturer. *p = *0.04*; ***p = *0.0008* and ****p < *0.0001*; ns, not significant. Results represent the average of three independent experiments.

Supplementary Figure 5

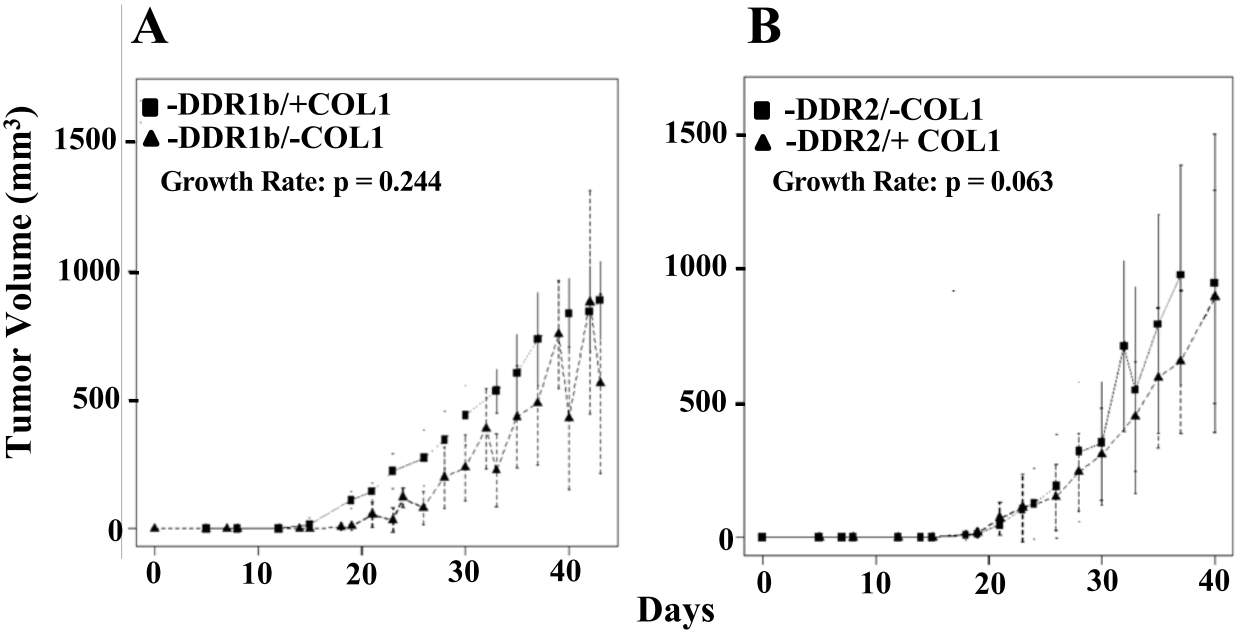

Suppl. Fig. 5 Volumes of -DDR/±COL1 tumours as a function of time. HT-DDR1b (**A**) and HT-DDR2 (**B**) cells were incubated for two days with or without DOX to repress or induce DDR expression, respectively. The +COL1 groups of cells were harvested and mixed with an ice-cold solution of rat-tail COL1 (2 mg/ml, final concentration), whereas the –COL groups of cells were suspended in serum free medium. Then, 1 x 10^6 cells/mouse were subcutaneously inoculated into mice. Number of mice for each group is provided in Suppl. Table 1. Tumors were measured every 2-3 days, and tumor volumes were calculated. Tumor growth rates were determined as described in the Methods section. Note: The -DDR/-COL1 groups here are the -DDR groups plotted in Fig. 2A-2B, and the -DDR/+COL1 groups are also plotted in Fig. 3A-3B.

Supplementary Figure 6

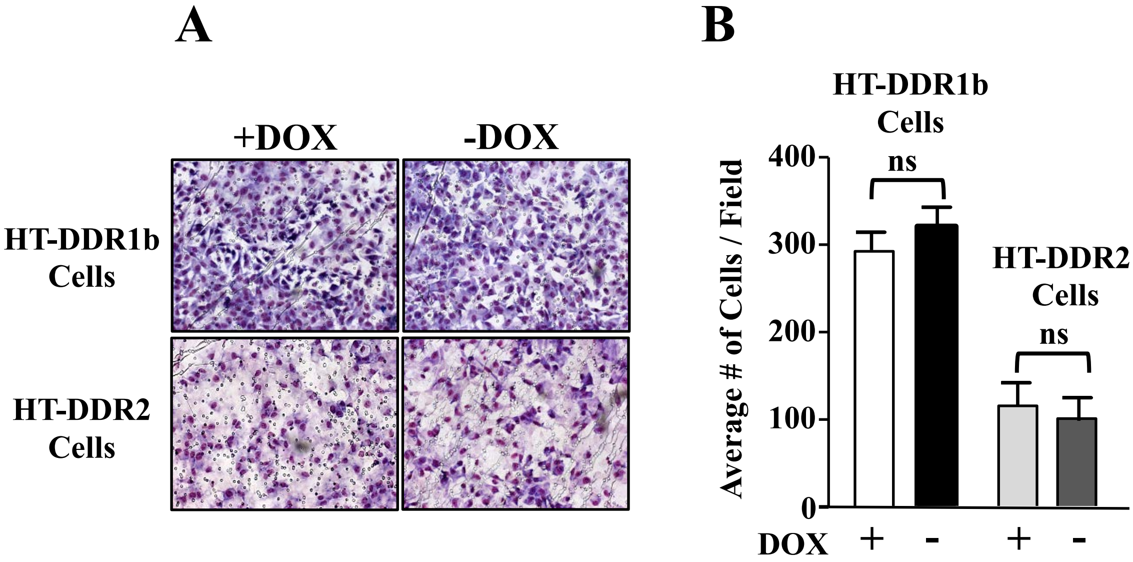

Suppl. Fig. 6. Effect of DDRs on HT1080 cell *in vitro* invasive ability.

(**A,B**) **Boyden chamber assay.** Corning® Transwell® culture inserts fitted with a polycarbonate filter were coated with Matrigel (50 µg/filter) and allowed to gel at 37 °C for 2 h. HT-DDR1b and HT-DDR2 cells, which were incubated 3 days in the presence or absence of DOX (±DOX), were harvested and 7.5 x 10^4 cells/filter in serum-free medium were plated on the Matrigel-coated inserts with or without DOX supplementation in triplicates. The inserts were placed on the wells, which were filled with 650 µl/well of complete medium ± DOX. After an 8-h incubation, the cells were fixed and stained with Diff-Quick. (**A**) Five pictures (four from upper/lower quadrant corners and one from the center) were taken at 20x magnification of each membrane and cells were counted (A, representative photographs). (**B**) The numbers of invading cells were then quantified. Results represent the average of three independent experiments. ns, not significant.

Supplementary Figure 6, cont'd

C

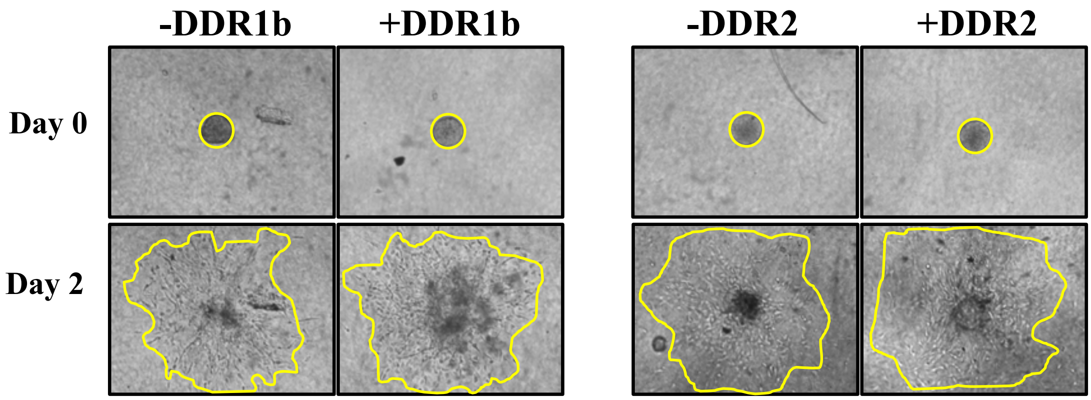

D

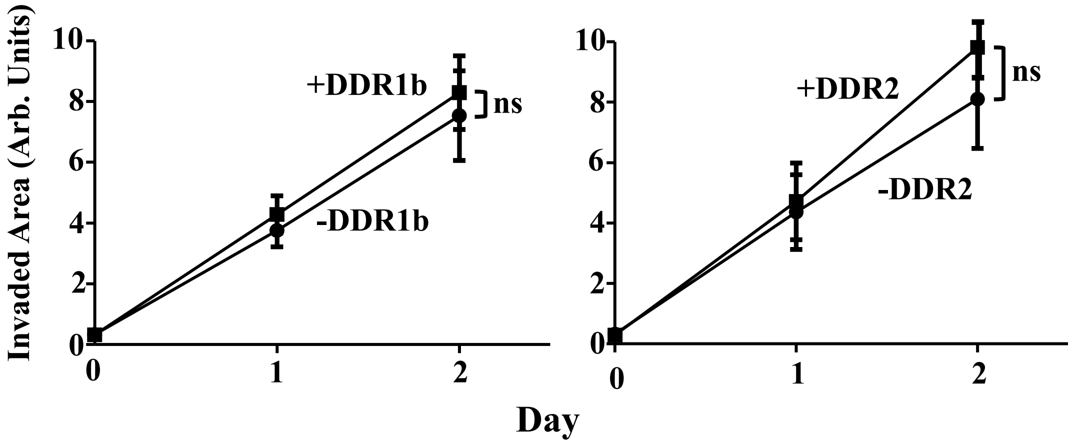

Suppl. Fig. 6, cont'd. Effect of DDRs on HT1080 cell *in vitro* invasive ability.

(**C,D**) **Spheroid invasion assay.** Cells (2.5 x 10^3 cells/well) were plated in the presence or absence of DOX in ultra-low attachment (ULA) 96-well plates in replicates of six. The cells were then incubated for 4 days at 37 °C, 5% CO_2 to allow spheroid formation. On day 4, DOX was removed from a subset of the +DOX group by diluting the DOX 64-fold. Then, 100 µl/well of neutralized COL1 (2 mg/ml final concentration) were added to the wells and allowed to polymerize for 1 h at 37 °C. After collagen polymerization, complete media (100 µl/well) were added on top of the COL1 gels. Cultures were photographed at 0, 1 and 2 days. (**C**) Representative photographs at 0 and 2 days. (**D**) Areas were quantified by tracing the edges of the invasive spheroids and calculating total pixels using ImageJ software. Replicate wells were then averaged and statistical analyses were performed as described in the Methods section. Results represent the average of three independent experiments.

Supplementary Figure 7

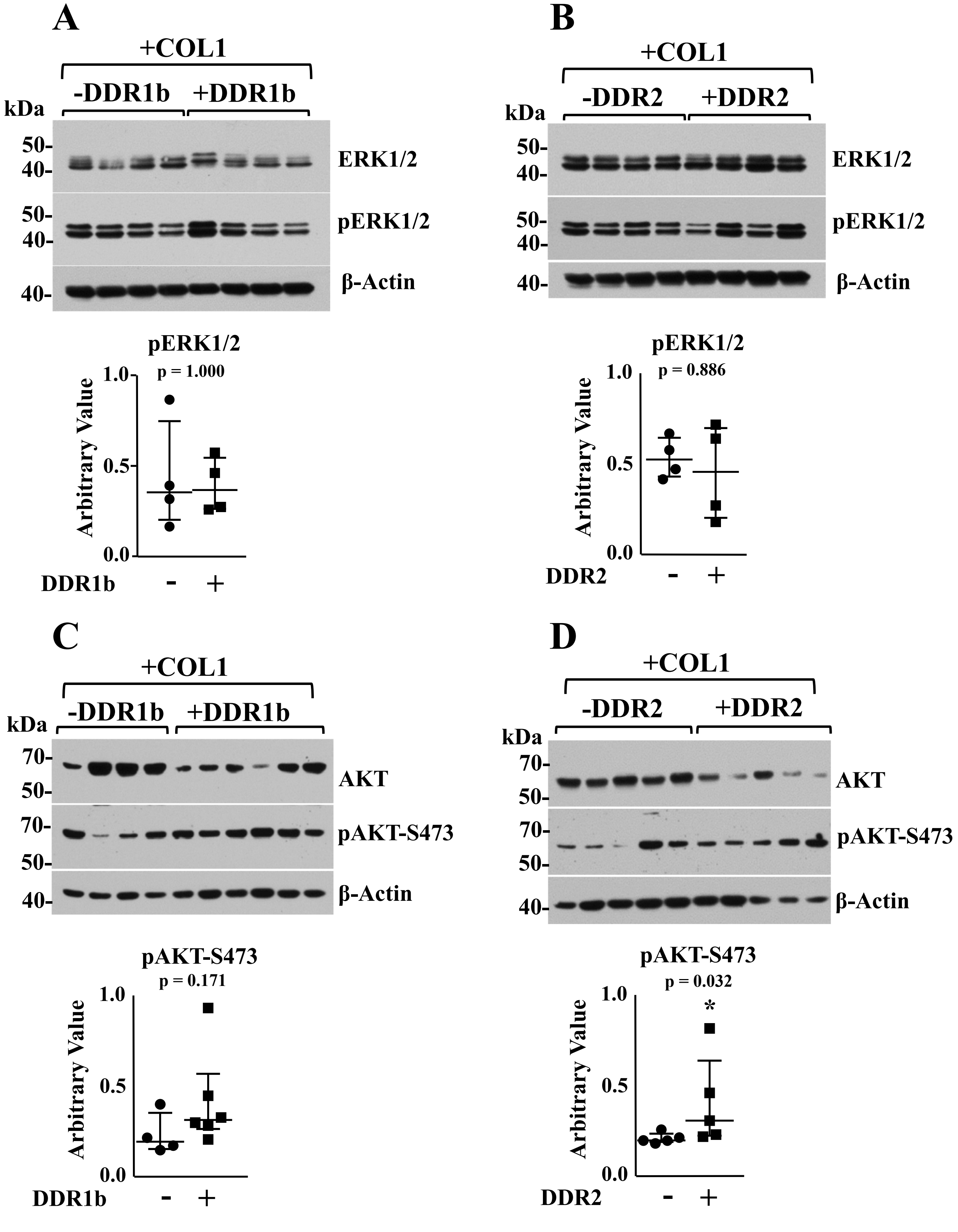

Supplementary Figure 7, cont'd

Suppl. Fig. 7. Impact of DDRs on ERK1/2 and AKT phosphorylation in ±DDR/+COL1 HT1080 tumours. Extracts of HT-DDR1b and HT-DDR2 tumours were resolved by reducing 10% SDS-PAGE followed by immunoblot analyses using antibodies to ERK1/2 and pERK1/2-T202/Y204 (**A**,**B**) and AKT and pAKT-S473 (**C**,**D**), described in Suppl. Table 2). The blots were then reprobed with antibodies for β-actin as loading control (lower panels). Each sample represents one mouse xenograft. Samples used for ERK1/2/pERK1/2: n = 4 in each ±DDR1b and ±DDR2 tumours; for AKT/pAKT: n = 4 in -DDR1b and n = 6 in +DDR1b tumours, and n = 5 both ±DDR2 tumours. Levels of pERK1/2 and pAKT were determined relative to total ERK1/2 and AKT, respectively. Scatter plots of pERK1/2 and pAKT show median with interquartile range where each dot represents an individual tumor. Mann-Whitney U test was performed for statistical analyses. *Asterisk indicates a non-specific band. Full-length blots are presented in Suppl. Figure 17.

Supplementary Figure 8

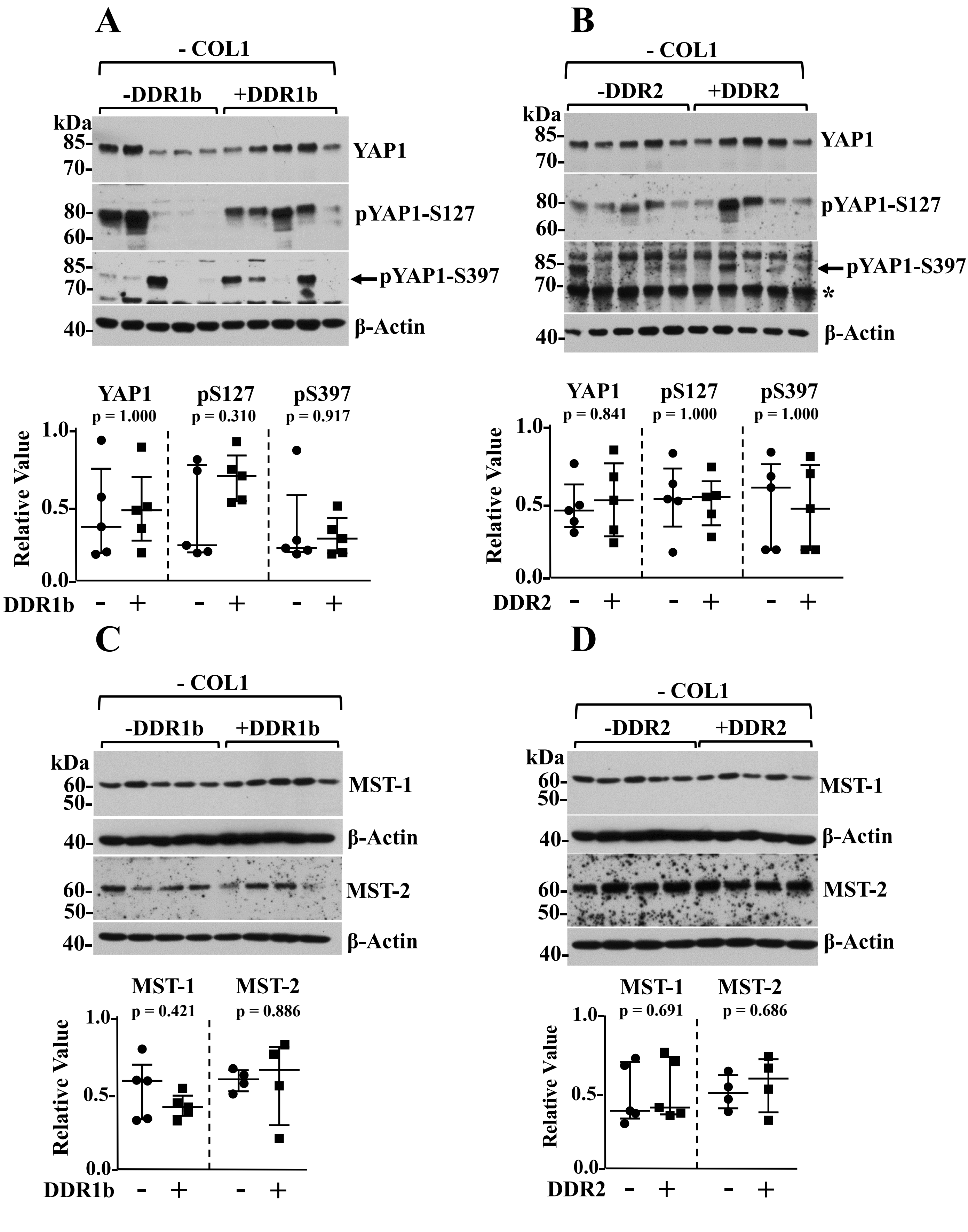

Supplementary Figure 8, cont'd

E

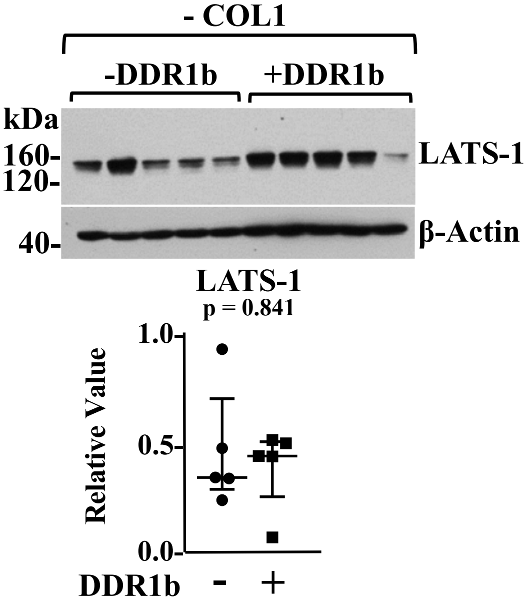

F

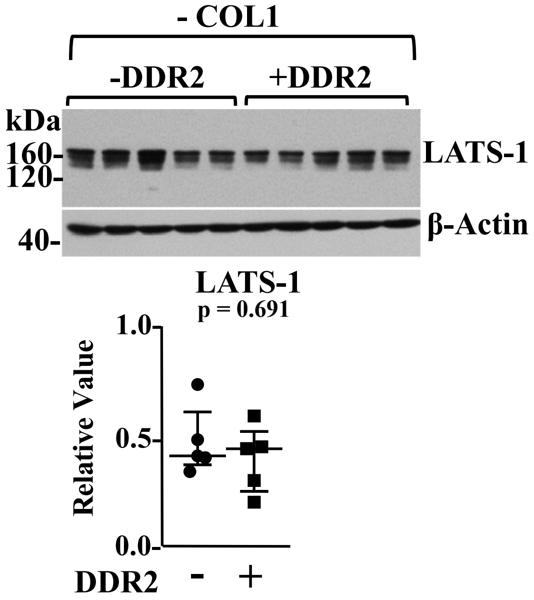

G

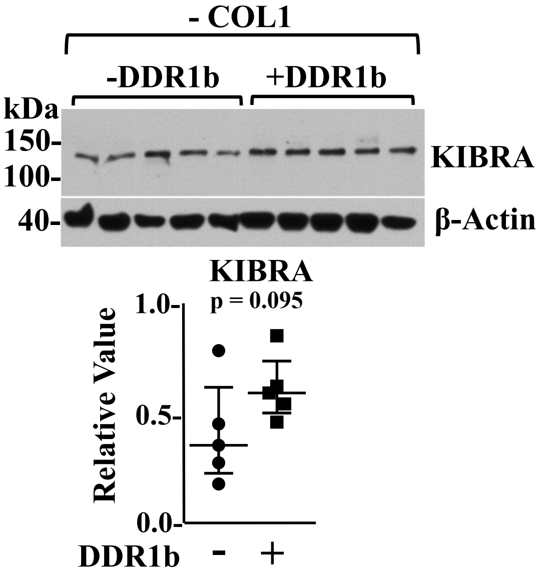

H

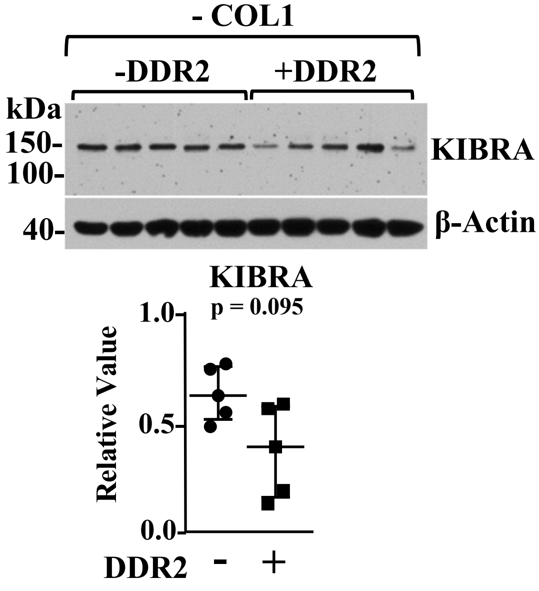

Supplementary Figure 8, cont'd

Suppl. Fig. 8. Impact of DDRs on Hippo pathway components in ±DDR/-COL1 HT1080 tumors. Extracts of HT-DDR1b and HT-DDR2 tumors (n = 5 for all tumor groups) were resolved by reducing SDS-PAGE followed by immunoblot analyses using antibodies to the indicated Hippo pathway components: YAP-1, pYAP1-S127, and pYAP1-S397 (**A**,**B**); MST1 and MST2 (**C**,**D**); LATS1 (**E**,**F**); and KIBRA (**G**,**H**). Blots were probed with the indicated antibodies and quantified as described in the Methods section. Note: in panels **A** and **B**, pYAP1-S127 and pYAP1-S397 were run in separate blots with the same tumor extract samples. Each blot was then stripped and reprobed for total YAP1. Then, relative levels of phosphorylated to total YAP1 levels were each performed in their respective blot. However, only one total YAP1 blot for DDR1b or DDR2 is shown. Scatter plots show median with interquartile range where each dot represents an individual tumor. Mann-Whitney U test was performed for statistical analyses. *Asterisk indicates a non-specific band. Full-length blots are presented in Suppl. Figure 18.

Supplementary Figure 9

Full-Length Blots of Figure 1*

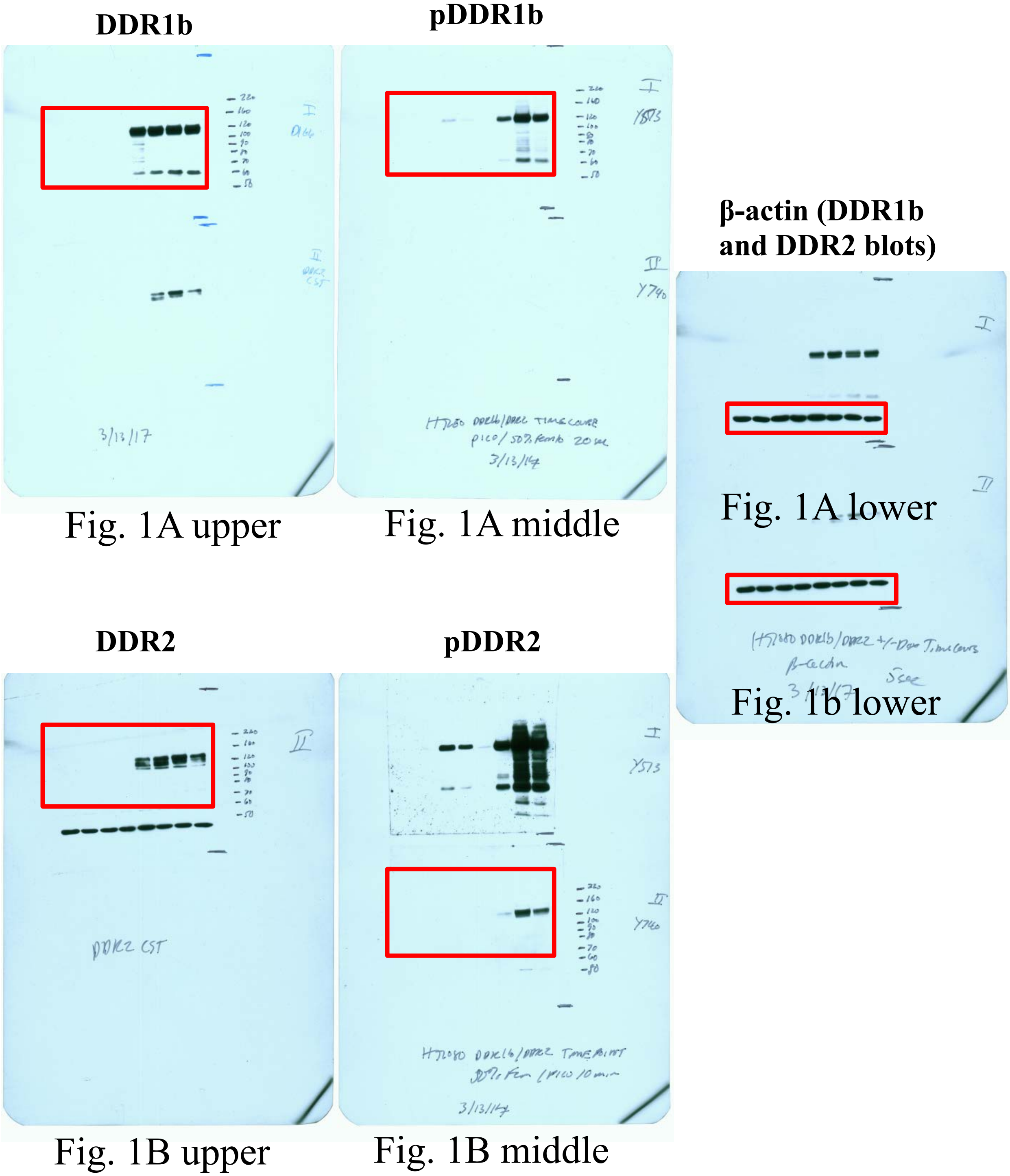

*Red rectangles represent the areas shown in the main Figures.

Supplementary Figure 10

Full-Length Blots of Figure 2*

DDR1b

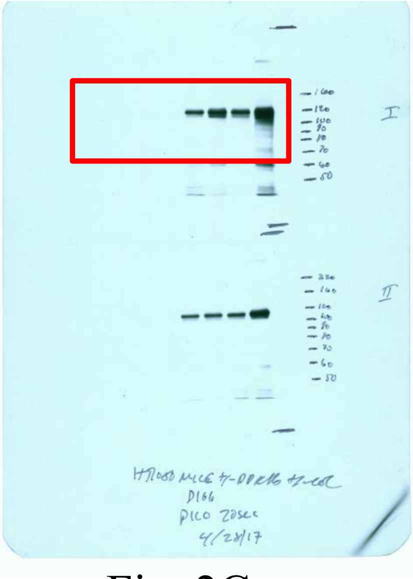

Fig. 2C upper

pDDR1b

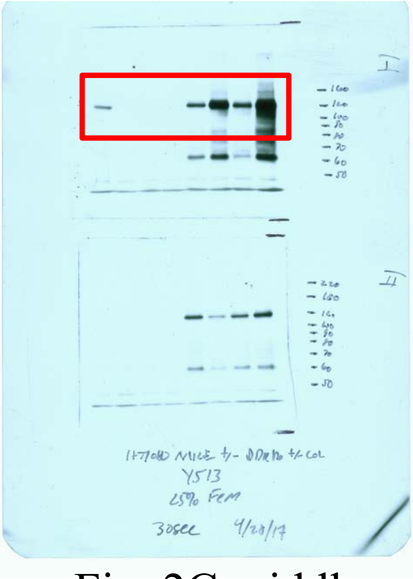

Fig. 2C middle

β-actin (DDR1b)

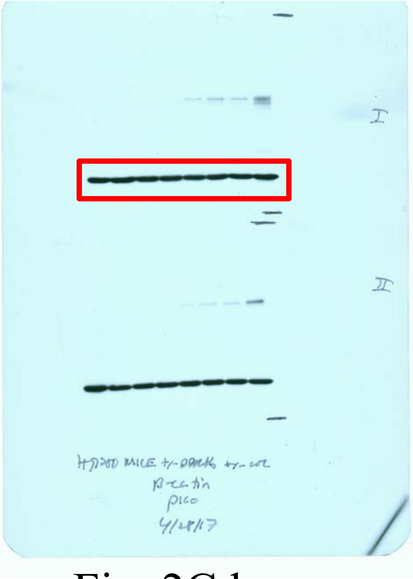

Fig. 2C lower

DDR2

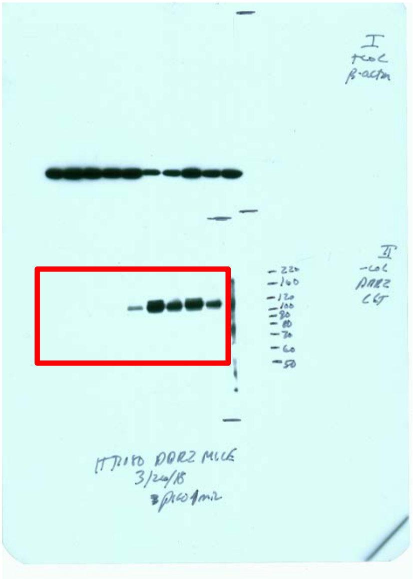

Fig. 2D upper

pDDR2

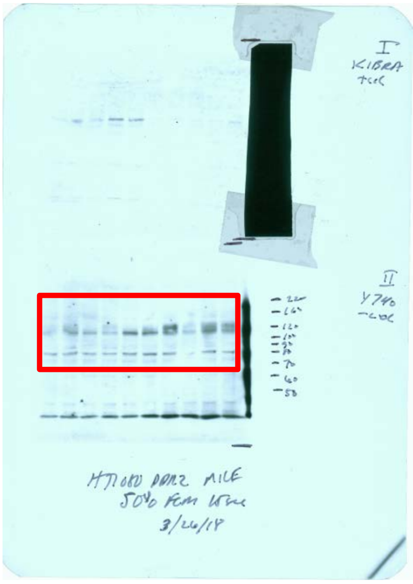

Fig. 2D middle

β-actin (DDR2)

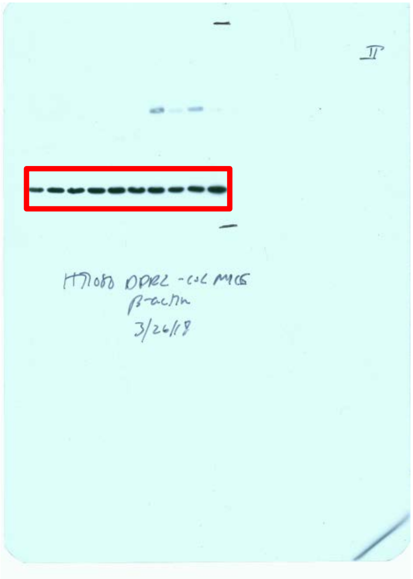

Fig. 2D lower

*Red rectangles represent the areas shown in the main Figures.

Supplementary Figure 11

Full-Length Blots of Figure 3*

DDR1b

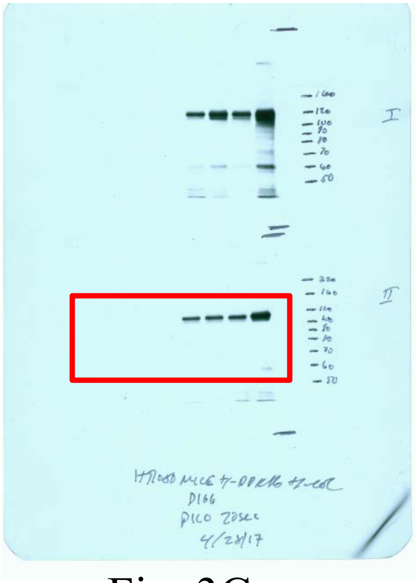

Fig. 3C upper

pDDR1b

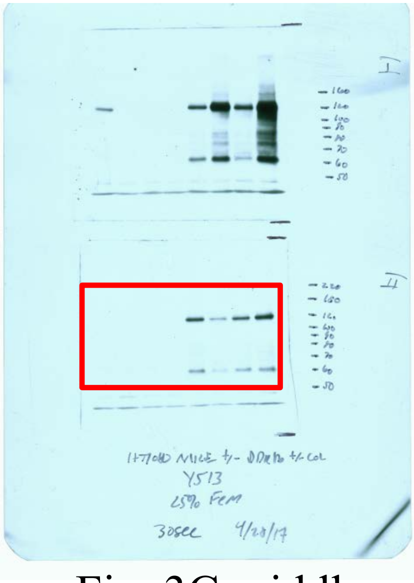

Fig. 3C middle

β-actin (DDR1b)

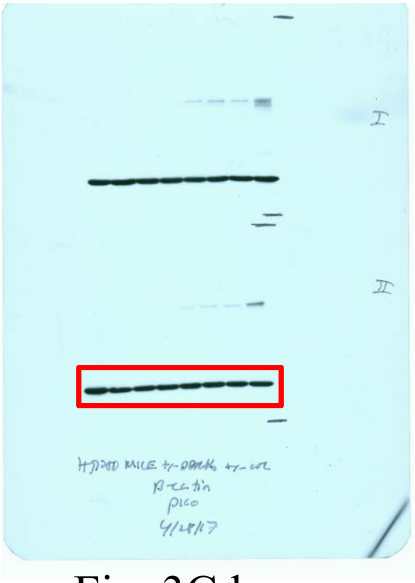

Fig. 3C lower

DDR2

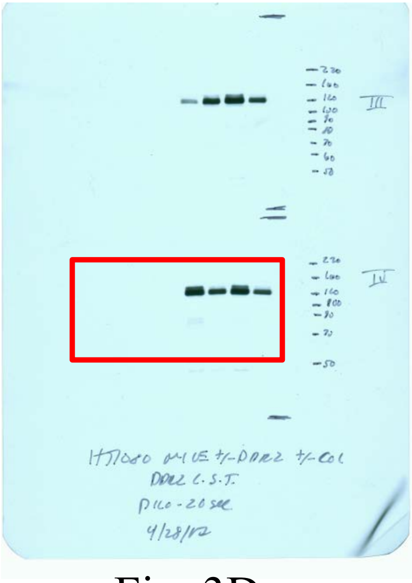

Fig. 3D upper

pDDR2

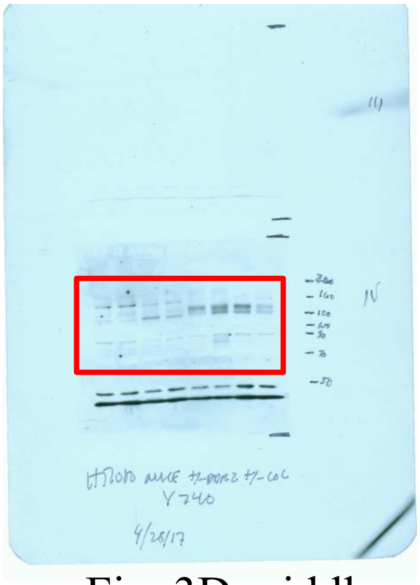

Fig. 3D middle

β-actin (DDR2)

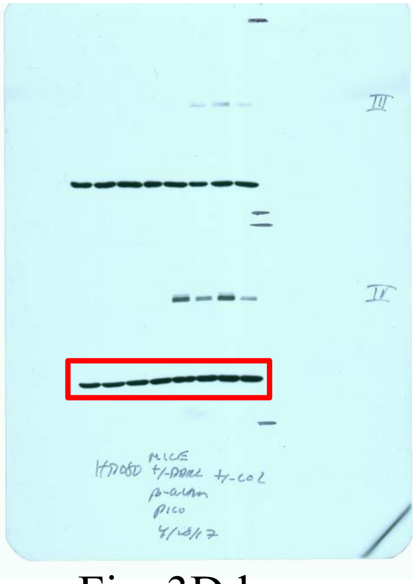

Fig. 3D lower

*Red rectangles represent the areas shown in the main Figures.

Supplementary Figure 12

Full-Length Blots of Figure 4*

DDR1b

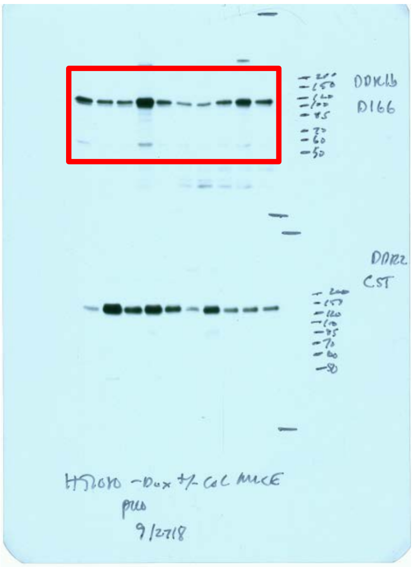

Fig. 4C upper

pDDR1b

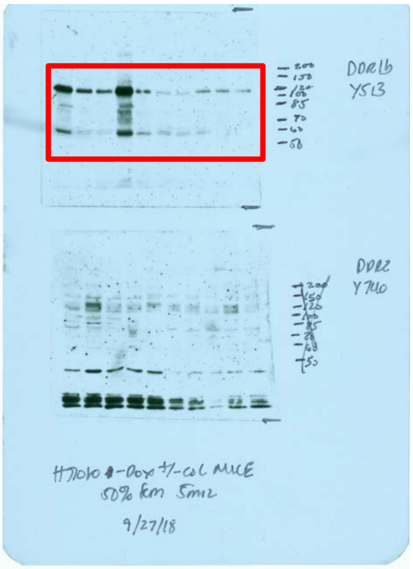

Fig. 4C middle

β-actin (DDR1b)

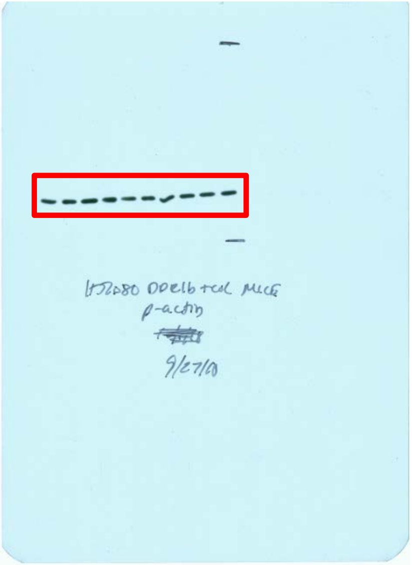

Fig. 4C lower

DDR2

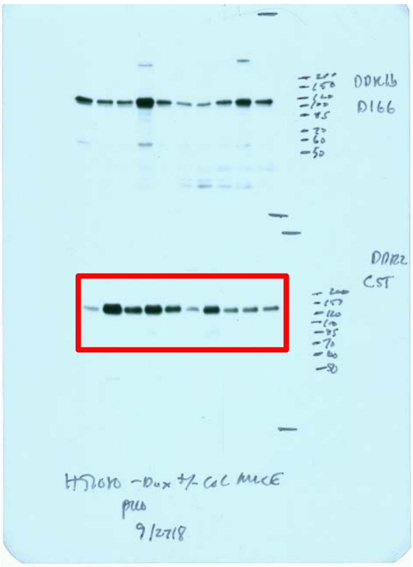

Fig. 4D upper

pDDR2

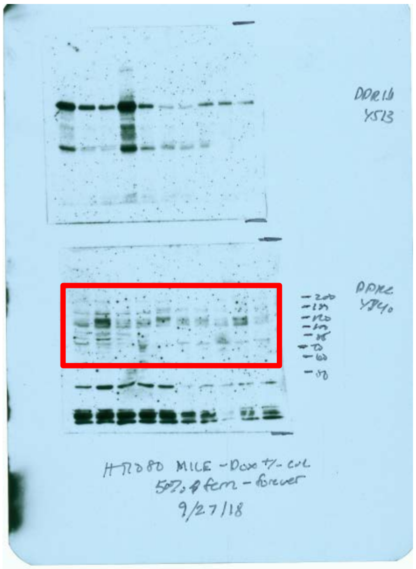

Fig. 4D middle

β-actin (DDR2)

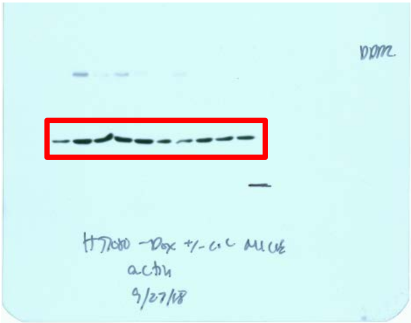

Fig. 4D lower

*Red rectangles represent the areas shown in the main Figures.

Supplementary Figure 12, cont'd

Full-Length Blots of Figure 4*

pDDR1b

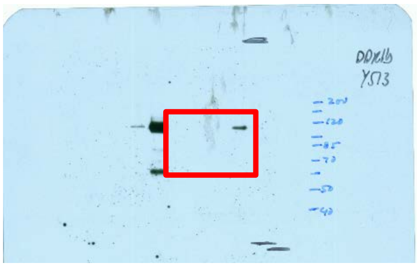

Fig. 4G upper

pDDR2

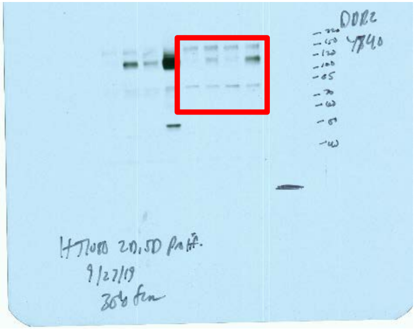

Fig. 4H upper

DDR1b

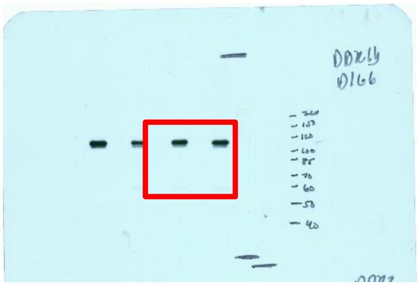

Fig. 4G middle

DDR2

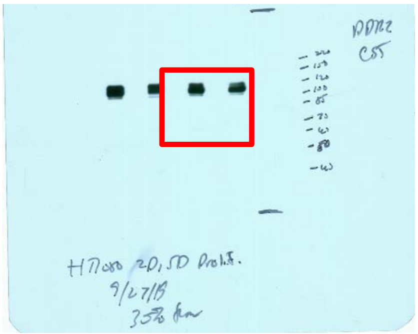

Fig. 4H middle

β-actin (DDR1b)

β-actin (DDR2)

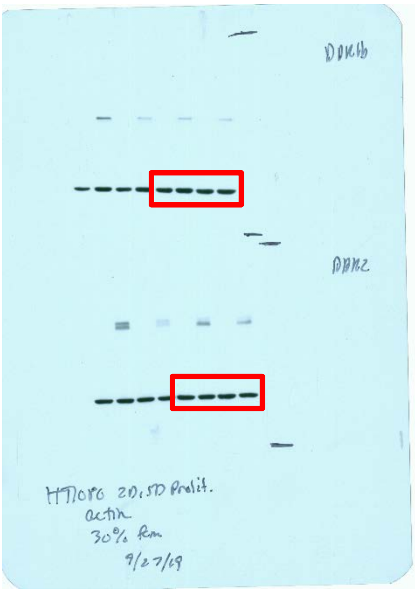

Fig. 4 G and H lower

*Red rectangles represent the areas shown in the main Figures.

Supplementary Figure 13

Full-Length Blots of Figure 6*

YAP (DDR1b)

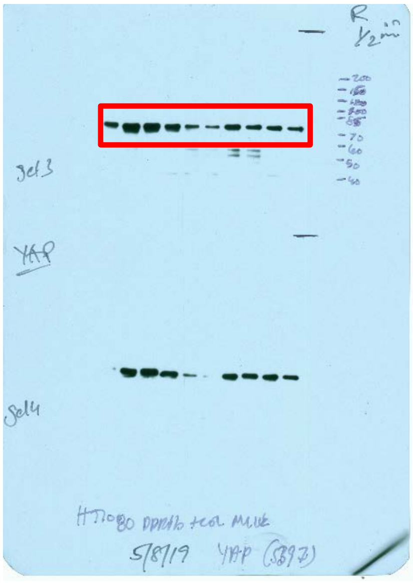

Fig. 6A upper

pYAP S397 (DDR1b)

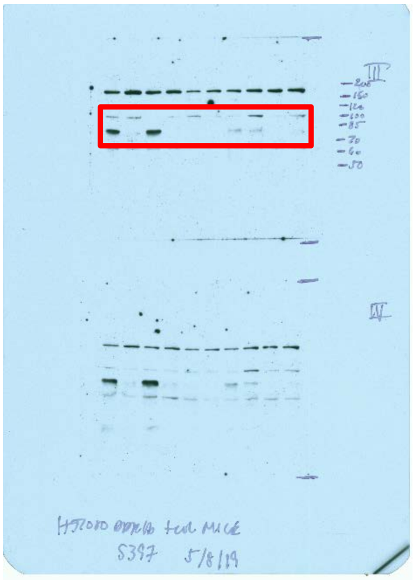

Fig. 6A third

pYAP S127 (DDR1b)

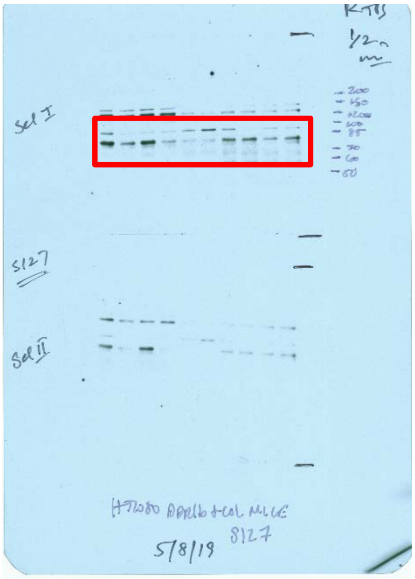

Fig. 6A second

β-actin YAP/S397 and S127 (DDR1b)

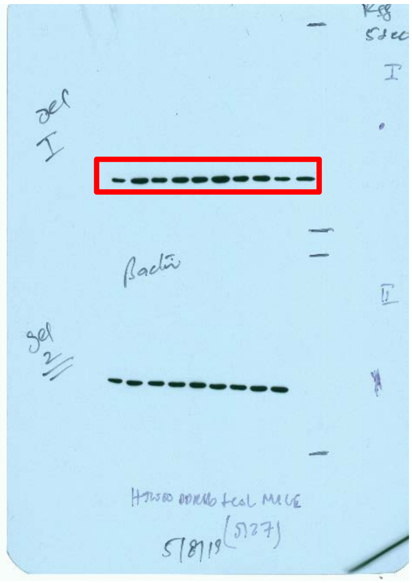

Fig. 6A lower

*Red rectangles represent the areas shown in the main Figures.

Supplementary Figure 13, cont'd

Full-Length Blots of Figure 6*

YAP (DDR2)

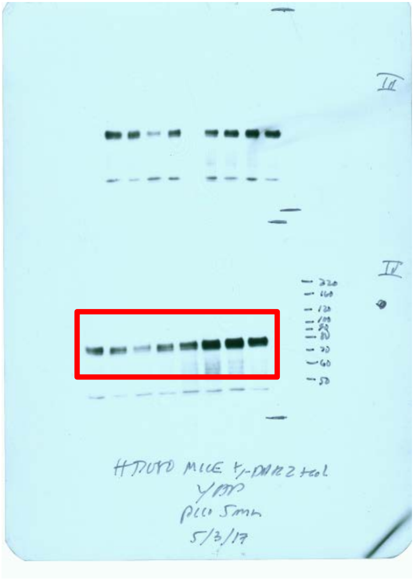

Fig. 6B upper

pYAP S397 (DDR2)

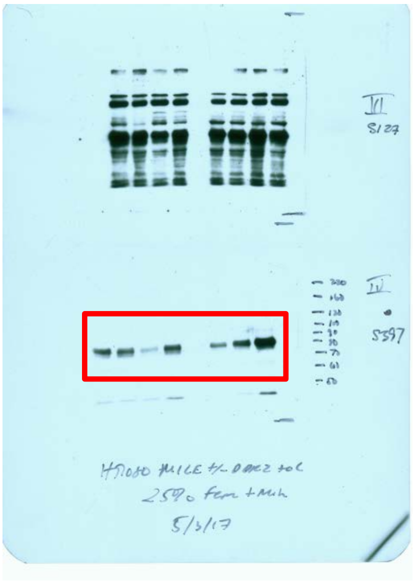

Fig. 6B third

pYAP S127 (DDR2)

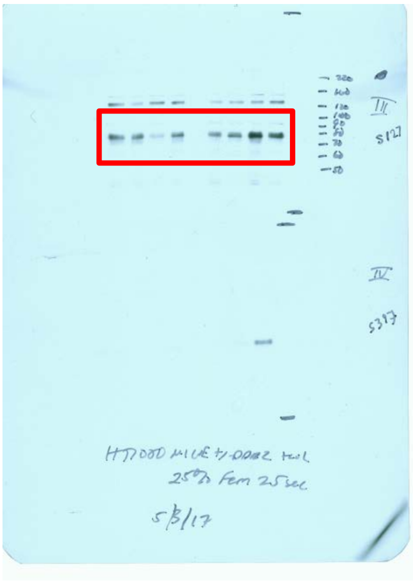

Fig. 6B second

β-actin YAP/S397 and S127 (DDR2)

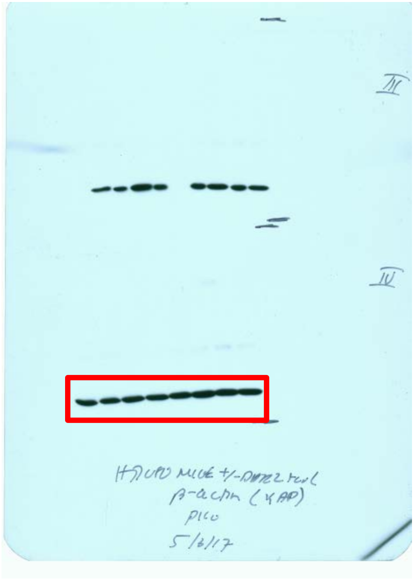

Fig. 6B lower

*Red rectangles represent the areas shown in the main Figures.

Supplementary Figure 13, cont'd

Full-Length Blots of Figure 6*

MST-1 (DDR1b)

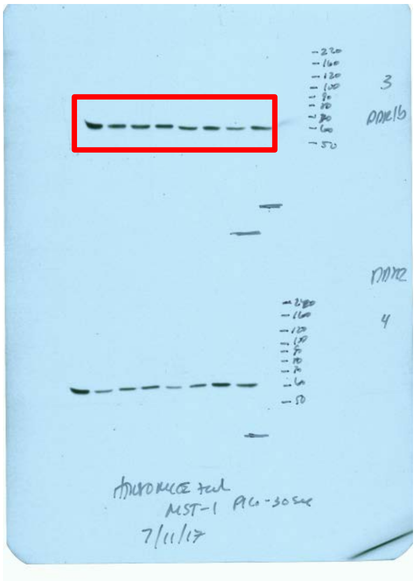

Fig. 6C upper

β-actin of MST-1 (DDR1b)

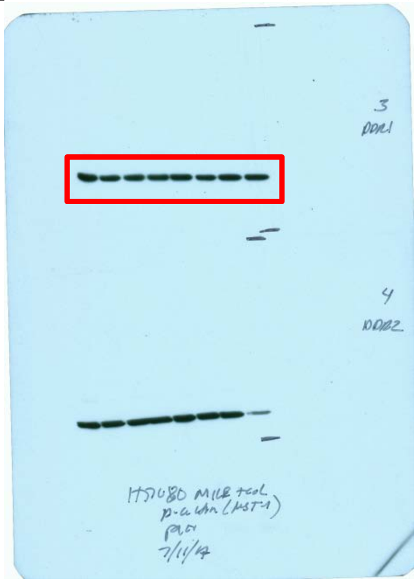

Fig. 6C second

MST-2 (DDR1b)

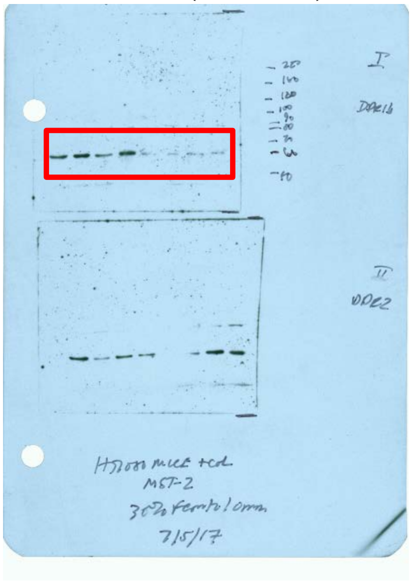

Fig. 6C third

β-actin of MST-2 (DDR1b)

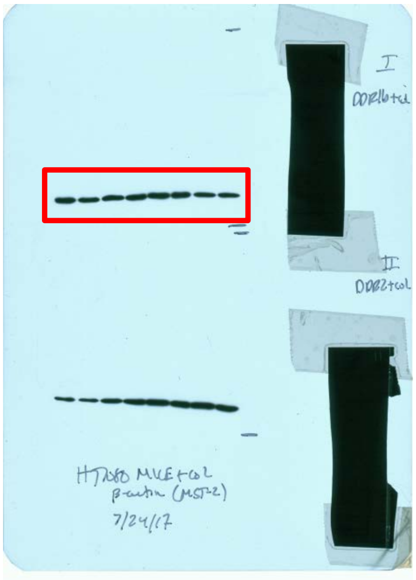

Fig. 6C lower

*Red rectangles represent the areas shown in the main Figures.

Supplementary Figure 13, cont'd

Full-Length Blots of Figure 6*

MST-1 (DDR2)

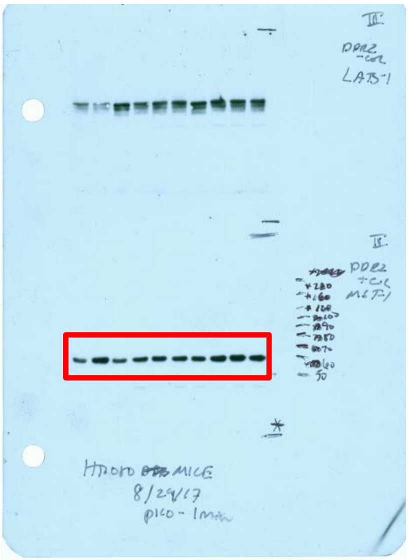

Fig. 6D upper

MST-2 (DDR2)

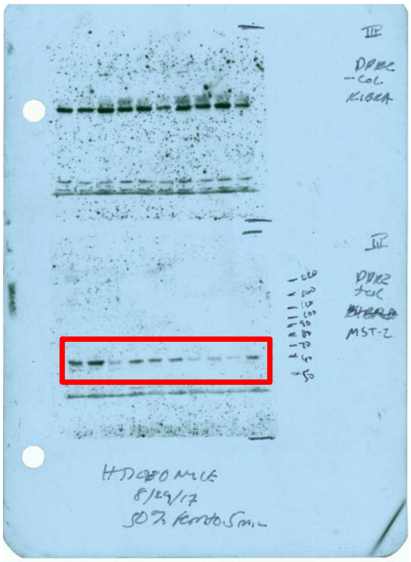

Fig. 6D second

β-actin of MST-1/MST-2 (DDR2)

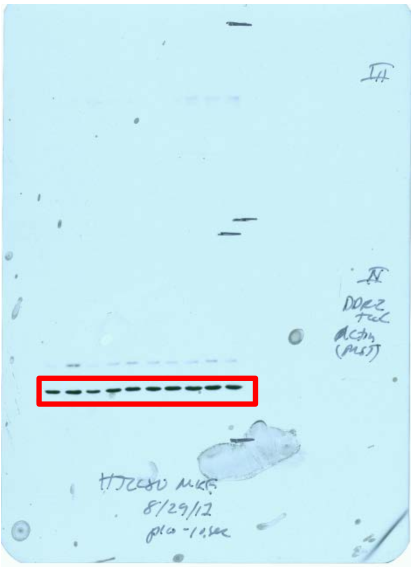

Fig. 6D lower

*Red rectangles represent the areas shown in the main Figures.

Supplementary Figure 13, cont'd

Full-Length Blots of Figure 6*

LATS-1 (DDR1b and DDR2)

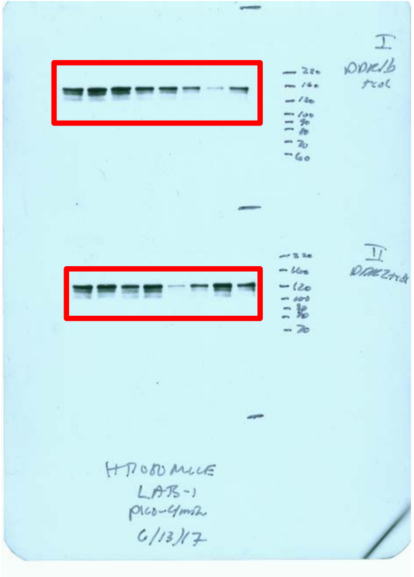

Fig. 6E upper

Fig. 6F upper

β-actin of LATS-1 (DDR1b and DDR2)

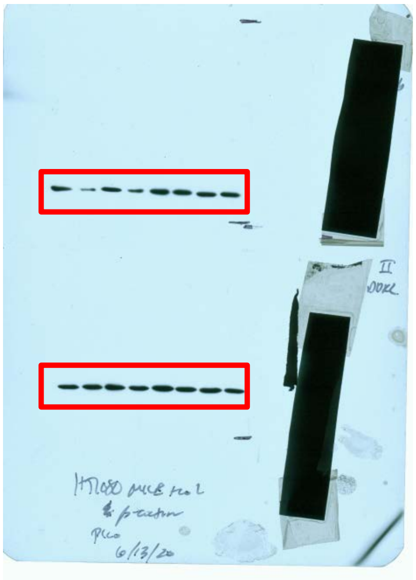

Fig. 6E lower

Fig. 6F lower

*Red rectangles represent the areas shown in the main Figures.

Supplementary Figure 13, cont'd

Full-Length Blots of Figure 6*

KIBRA (DDR1b)

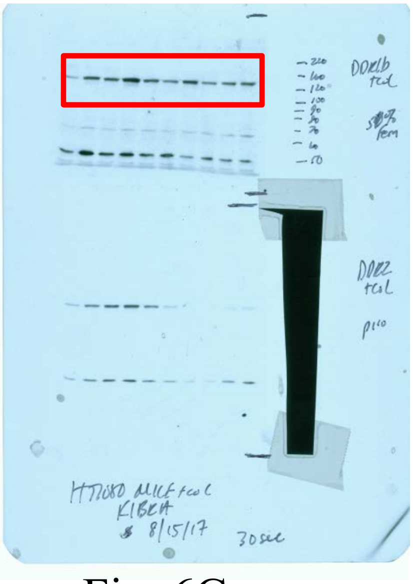

Fig. 6G upper

β-actin of KIBRA (DDR1b)

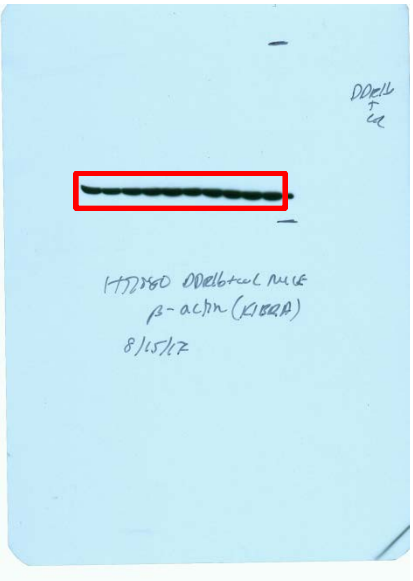

Fig. 6G lower

KIBRA (DDR2)

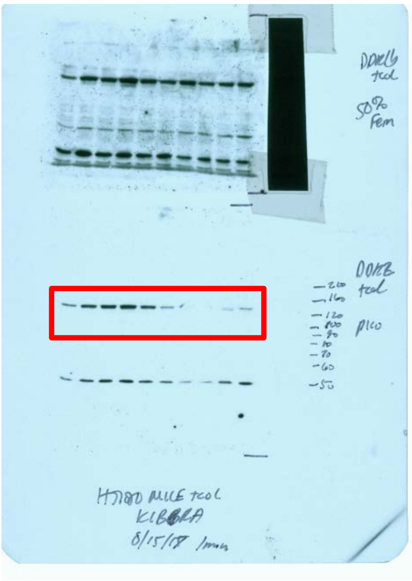

Fig. 6H upper

β-actin of KIBRA (DDR2)

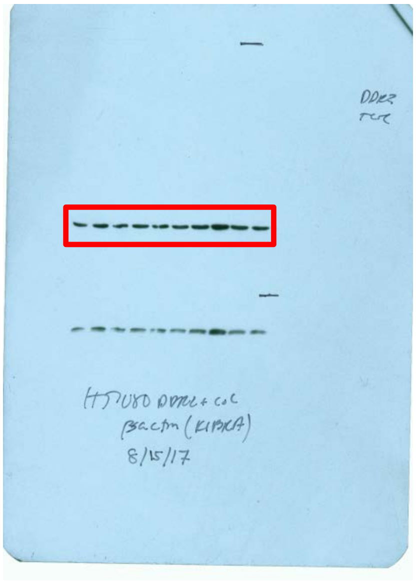

Fig. 6H lower

*Red rectangles represent the areas shown in the main Figures.

Supplementary Figure 14

Full-Length Blots of Suppl. Figure 1*

DDR1b

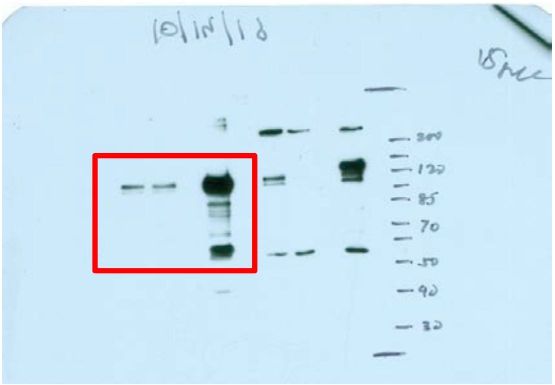

Fig. 1A upper

DDR2

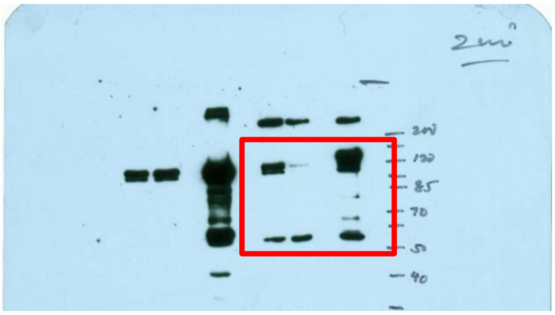

Fig. 1B upper

β-actin DDR1

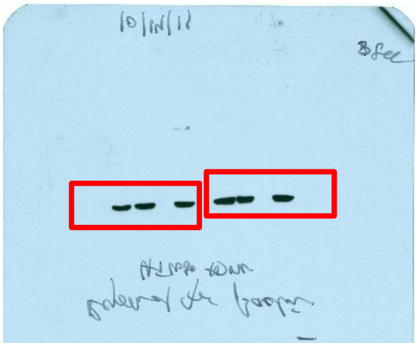

β-actin DDR2

*Red rectangles represent the areas shown in the corresponding Suppl. Figure

Supplementary Figure 15

Full-Length Blots of Suppl. Figure 2*

DDR1b **pDDR1b** **β-actin**

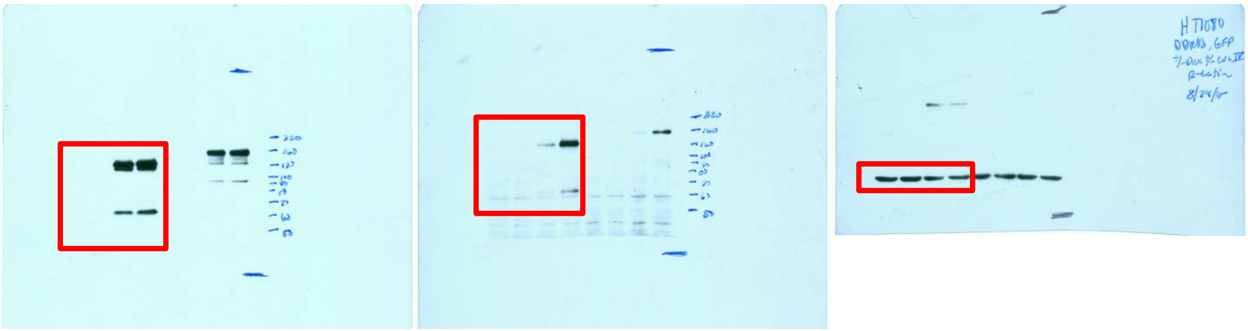

Upper panel Middle panel Lower panel

*Red rectangles represent the areas shown in the corresponding Suppl. Figure

Supplementary Figure 16

Full-Length Blots of Suppl. Figure 3*

DDR1b

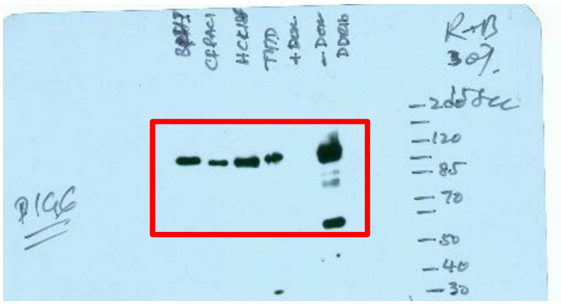

Fig. 3A upper

DDR2

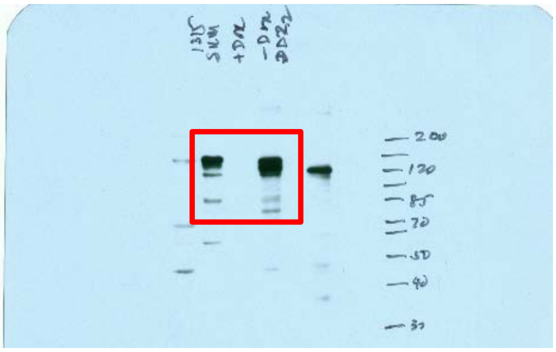

Fig. 3B upper

β-actin

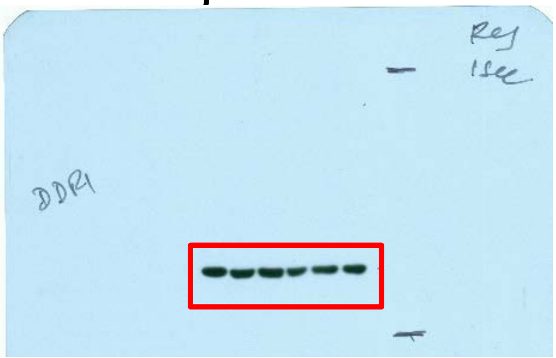

Fig. 3A lower

β-actin

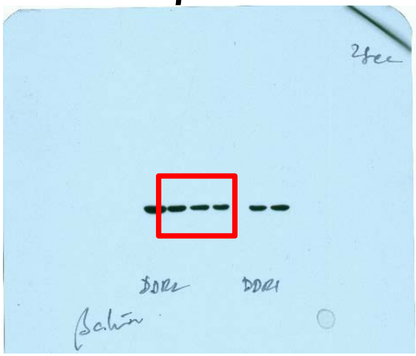

Fig. 3B lower

*Red rectangles represent the areas shown in the corresponding Suppl. Figure

Supplementary Figure 17

Full-Length Blots of Suppl. Figure 7*

Total ERK1/2 (DDR1b)

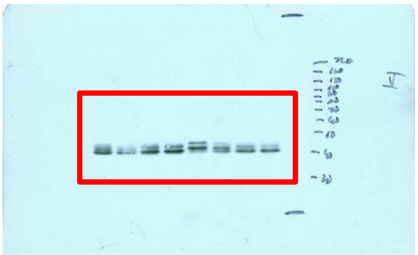

Fig. 7A upper

pERK1/2 (DDR1b)

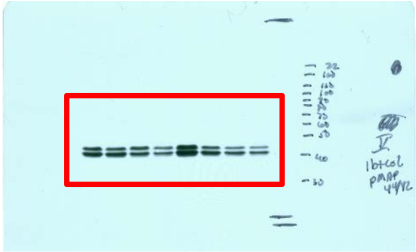

Fig. 7A middle

β-actin (DDR1b)

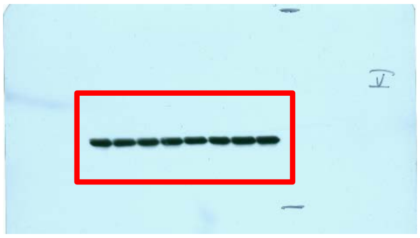

Fig. 7A lower

Total ERK1/2 (DDR2)

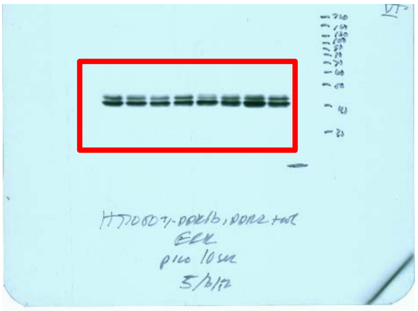

Fig. 7B upper

pERK1/2 (DDR2)

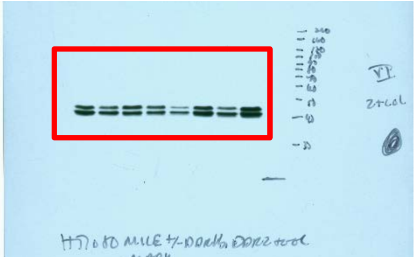

Fig. 7B middle

β-actin (DDR2)

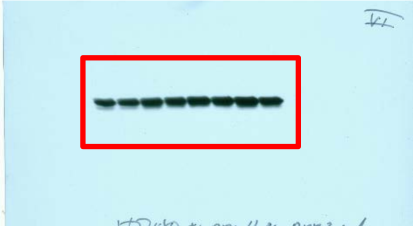

Fig. 7B lower

*Red rectangles represent the areas shown in the corresponding Suppl. Figure

Supplementary Figure 17, cont'd

Full-Length Blots of Suppl. Figure 7*

Total AKT (DDR1b)

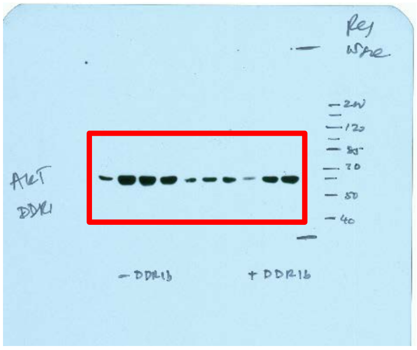

Fig. 7C upper

Total AKT (DDR2)

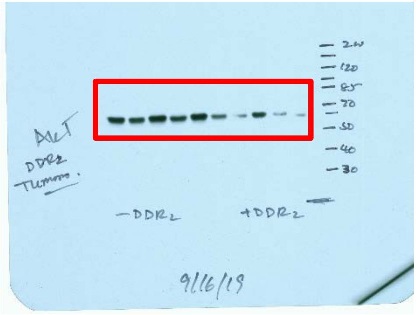

Fig. 7D upper

pAKT (DDR1b)

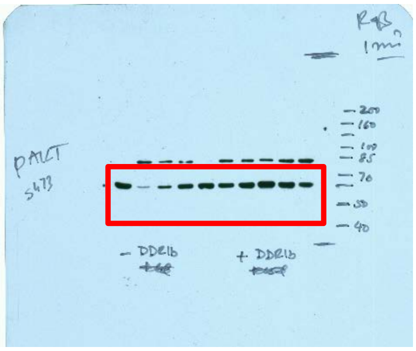

Fig. 7C middle

pAKT (DDR2)

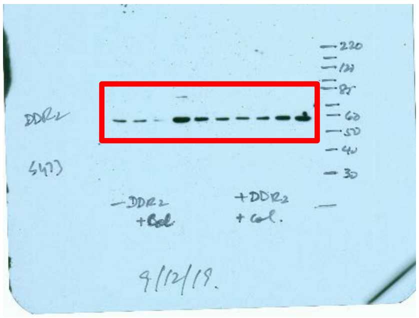

Fig. 7D middle

β-actin (DDR1b)

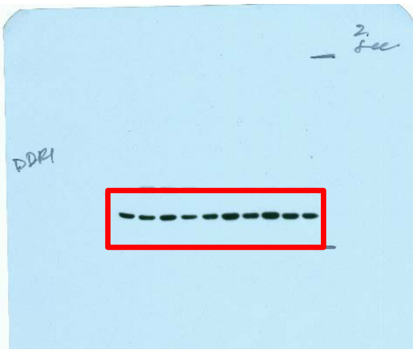

Fig. 7C lower

β-actin (DDR2)

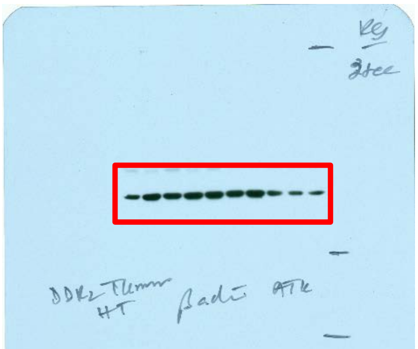

Fig. 7D lower

*Red rectangles represent the areas shown in the corresponding Suppl. Figure

Supplementary Figure 18

Full-Length Blots of Suppl. Figure 8*

YAP (DDR1b)

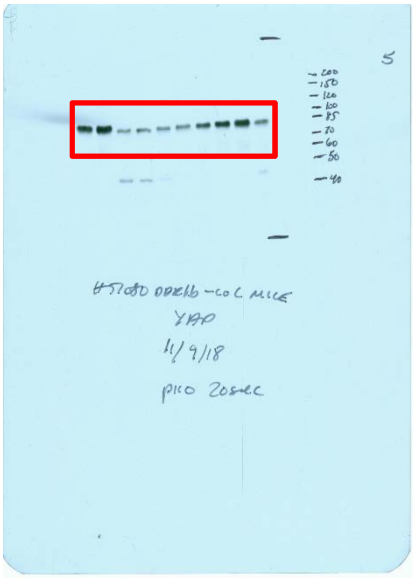

Suppl. Fig. 8A upper

pYAP S127 (DDR1b)

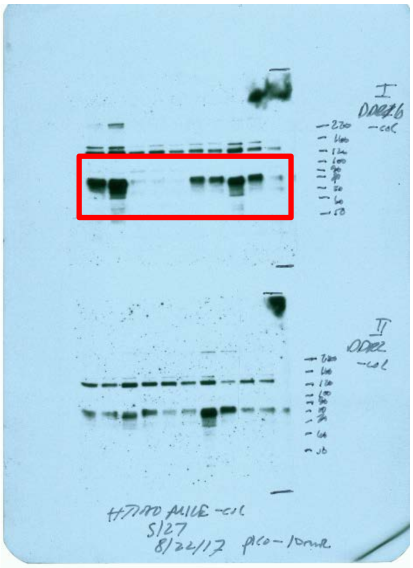

Suppl. Fig. 8A second

pYAP S397 (DDR1b)

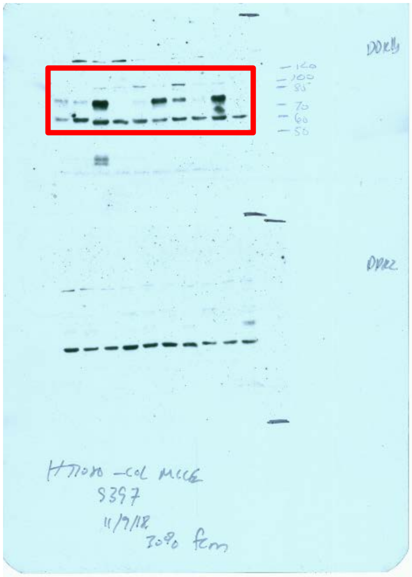

Suppl. Fig. 8A third

β-actin YAP/S397 and S127 (DDR1b)

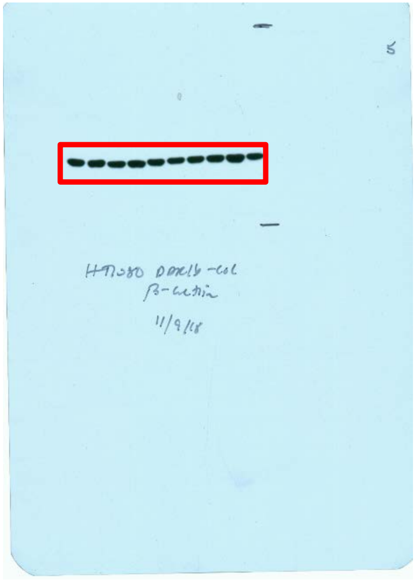

Suppl. Fig. 8A lower

*Red rectangles represent the areas shown in the corresponding Suppl. Figure

Supplementary Figure 18, cont'd

Full-Length Blots of Suppl. Figure 8*

YAP (DDR2)

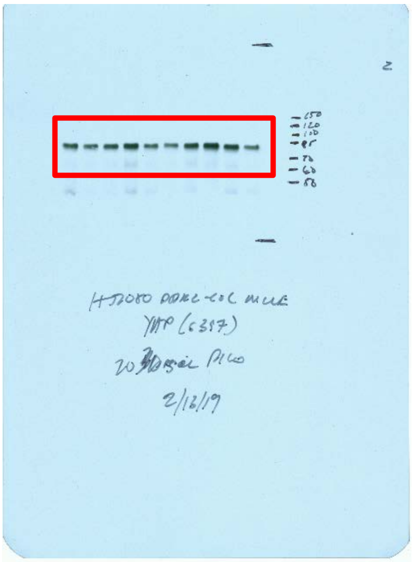

Suppl. Fig. 8B upper

pYAP S127 (DDR2)

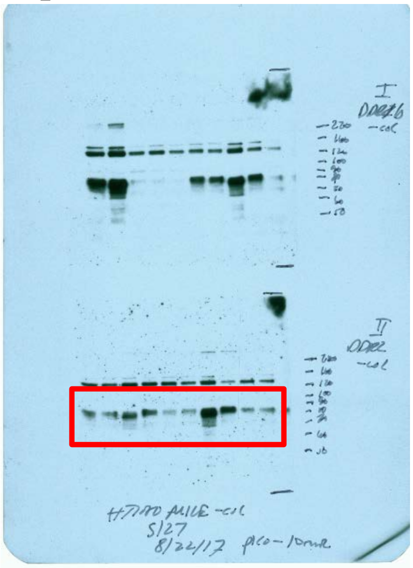

Suppl. Fig. 8B second

pYAP S397 (DDR2)

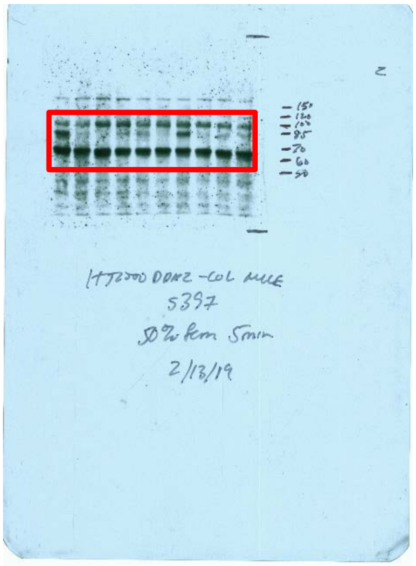

Suppl. Fig. 8B third

β-actin YAP/S397 and S127 (DDR2)

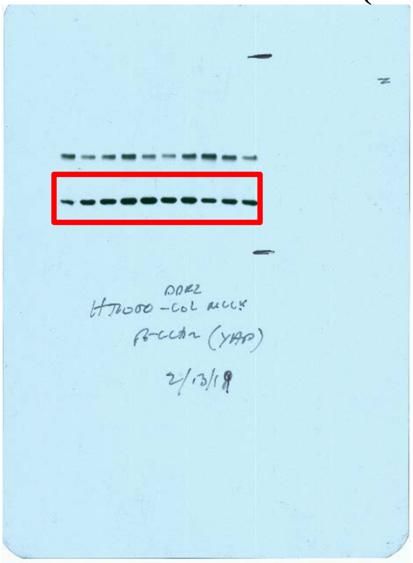

Suppl. Fig. 8B lower

*Red rectangles represent the areas shown in the corresponding Suppl. Figure

Supplementary Figure 18, cont'd

Whole Blots of Suppl. Figure 8*

MST-1 (DDR1b and DDR2)

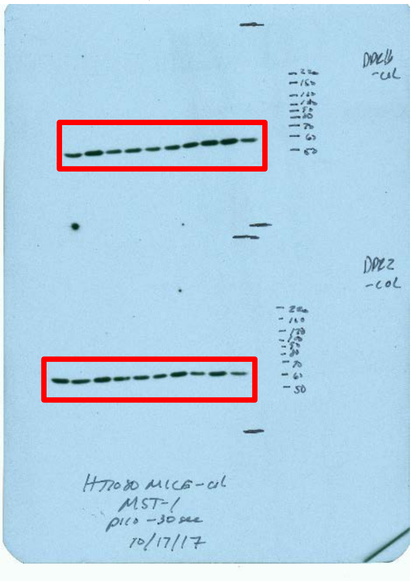

Suppl. Fig. 8C upper

Suppl. Fig. 8D upper

β-actin of MST-1 (DDR1band DDR2)

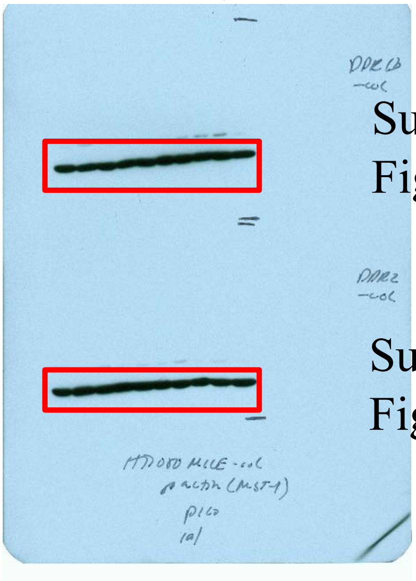

Suppl. Fig. 8C second

Suppl. Fig. 8D second

MST-2 (DDR1b and DDR2)

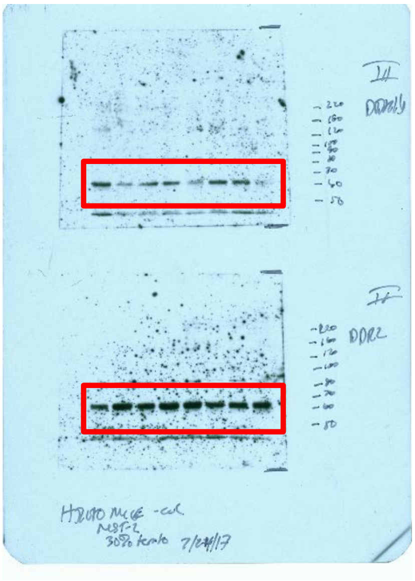

Suppl. Fig. 8C third

Suppl. Fig. 8D third

β-actin of MST-2 (DDR1b and DDR2)

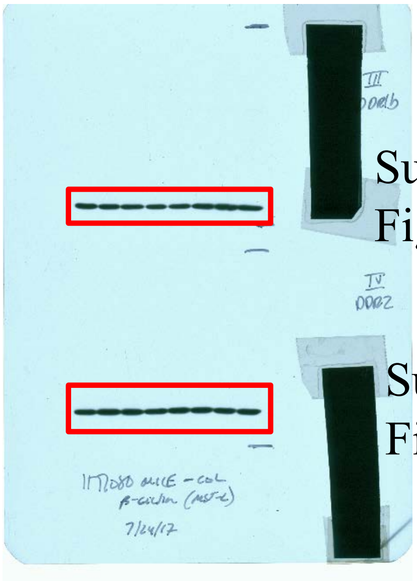

Suppl. Fig. 8C lower

Suppl. Fig. 8D lower

*Red rectangles represent the areas shown in the corresponding Suppl. Figure

Supplementary Figure 18, cont'd

Full-Length Blots of Suppl. Figure 8*

LATS-1 (DDR1b)

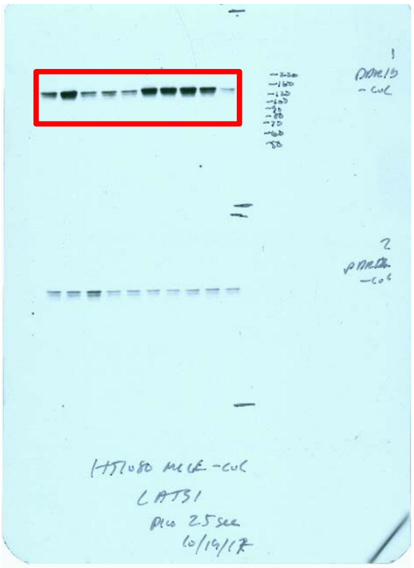

Suppl. Fig. 8E upper

LATS-1 (DDR2)

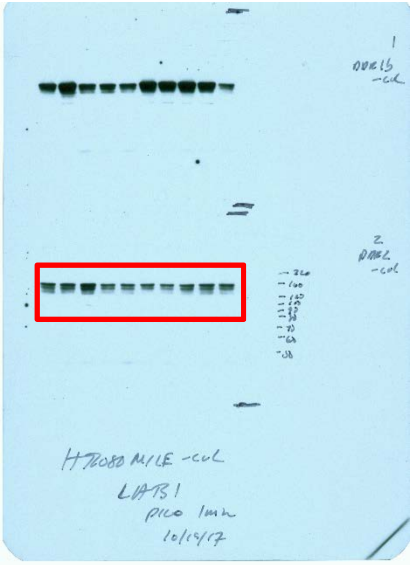

Suppl. Fig. 8F upper

β-actin of LATS-1 (DDR1b and DDR2)

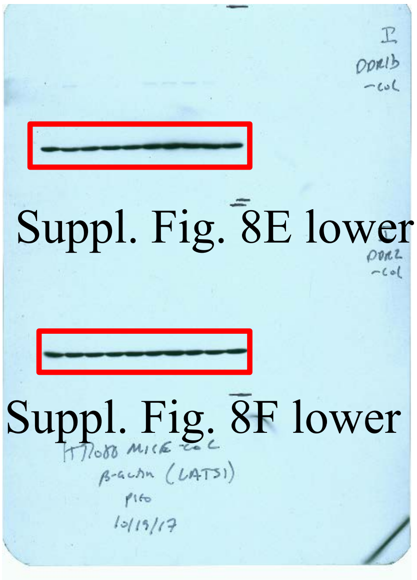

Suppl. Fig. 8E lower

Suppl. Fig. 8F lower

KIBRA (DDR1b)

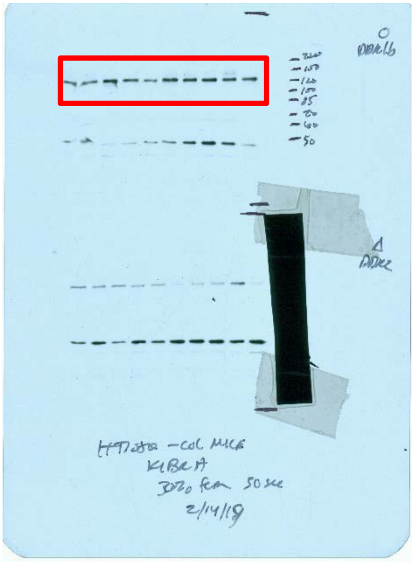

Suppl. Fig. 8G upper

KIBRA (DDR2)

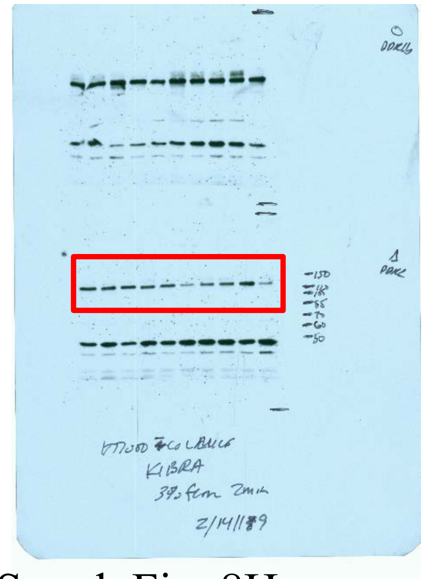

Suppl. Fig. 8H upper

β-actin of KIBRA (DDR1b and DDR2)

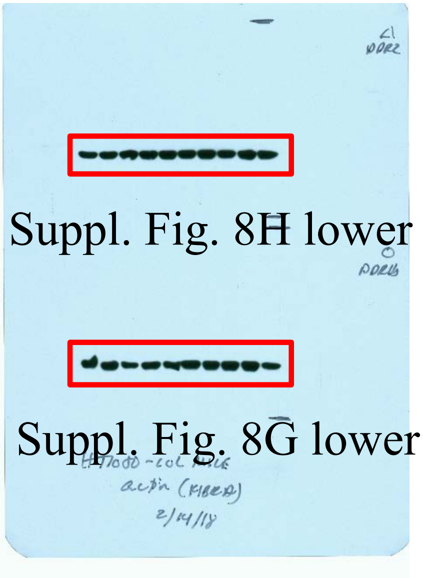

Suppl. Fig. 8H lower

Suppl. Fig. 8G lower

*Red rectangles represent the areas shown in the corresponding Suppl. Figure

Supplementary Table 1

Experimental Group (G) # of Mice/Group (n)		Tumor Growth Rate		Tumor Size		Tumor Latency	
G1	G2	Faster rate	*p*	Larger size	p	Shorter latency	*p*
+DDR1 n=11	-DDR1 n=9	ND	ns	ND	ns	ND	ns
+DDR1/+COL1 n=6	-DDR1/+COL1 n=6	+DDR1/+COL1	***	+DDR1/+COL1	*	+DDR1/+COL1	*
+DDR1/+COL1 n=6	+DDR1 n=11	+DDR1/+COL1	*	ND	ns	ND	ns
-DDR1/+COL1 n=6	-DDR1 n=9	ND	ns	ND	ns	ND	ns
-DDR2 n=11	+DDR2 n=12	ND	ns	ND	ns	ND	ns
+DDR2/+COL1 n=6	-DDR2/+COL1 n=6	+DDR2/+COL1	***	+DDR2/+COL1	*	+DDR2/+COL1	***
+DDR2/+COL1 n=6	+DDR2 n=12	+DDR2/+COL1	**	ND	ns	ND	ns
-DDR2/+COL1 n=6	-DDR2 n=11	ND	ns	ND	ns	ND	ns

Supp. Table 1. Summary of s.c. tumor growth studies. Tumors of HT-DDR1b or HT-DDR2 cells, with (+) or without (-) DDR expression that were inoculated with (+) or without COL1 were evaluated for tumor volumes as a function of time. The number of mice (n) for each experimental groups is indicated. Statistical analyses for tumor growth rate, tumor size at time of sacrifice, and tumor latency (the time to reach a size of 1,000 mm^3) were described in the Methods section. * $p = < 0.5$, ** $p = < 0.01$, *** $p = < 0.001$, ns = not significant. ND: no differences between the groups.

Supplementary Table 2

LIST OF ANTIBODIES

Antigen	Company	Cat #	Species	Dilution
**DDR1**	CST	5583 (D1G6)	Rabbit mAb	Western Blot (WB): 1:2000
**pDDR1b (Tyr513)**	CST	14531	Rabbit mAb	WB: 1:2000
**DDR2**	CST	12133	Rabbit mAb	WB: 1:2000
**pDDR2 (Tyr740)**	CST	25382	Rabbit mAb	WB: 1:2000
**LATS1**	CST	3477	Rabbit mAb	WB: 1:2000
**MST1**	CST	3682	Rabbit pAb	WB: 1:2000
**MST2**	CST	3952	Rabbit pAb	WB: 1:2000
**YAP1**	CST	12395	Mouse mAb	WB: 1:2000<br>IHC: 1:500
**pYAP1 (Ser127)**	CST	13008	Rabbit mAb	WB: 1:2000
**pYAP1 (Ser397)**	CST	13619	Rabbit mAb	WB: 1:2000
**KIBRA**	CST	8774	Rabbit pAb	WB: 1:2000
**β-actin**	Sigma	a5441	Mouse mAb	WB: 1:5000
**ERK1/2**	CST	4695	Rabbit mAb	WB: 1:2000
**pERK1/2 (Thr202/Tyr204)**	CST	4370	Rabbit mAb	WB: 1:2000
**AKT**	CST	9272	Rabbit pAb	WB: 1:2000
**pAKT (Ser473)**	CST	4060	Rabbit pAb	WB: 1:2000

Supplementary Table 3

LIST OF PCR PRIMERS

Gene	Forward	Reverse
**Human CTGF**	5’ – GCTCGGTAT GTCTTCATGCTG – 3’	5’ – GAAGCTGACCTGGAAGAGAAC – 3’
**Human CYR61**	5’ – GGGATTTCTTGGTCTTGCTG– 3’	5’ – CCAATGACAACCCTGAGTGC – 3’
**Human AXL**	5’ – GTCAAATTCCTTCATGCAGACC – 3’	5’ – TGGCTGTGAAGACGATGAAG – 3’
**Human GAPDH**	5’ – TGTAGT TGAGGT AATGAAGGG – 3’	5’ – ACATCGCTCAGACACCAT G – 3’
**Human Alu**	5’ – GTCAGGAGATCGAGACCATCCC– 3’	5’ – TCCTGCCTCAGCCTCCCAAG – 3’
**Mouse Actin**	5’ – GACTCATCGTACTCCTGCTTG – 3’	5’ – GATTACTGCTCTTGGCTCCTA G – 3’
**DDR1a/b**	5’ – GGAAGAGCGATGAGAGGTGT– 3’	5’ – CCTGATCCCTCGGGCCTAA– 3’
**DDR1c**	5’ – AATGCCAGCTTCTCCTTGTTC– 3’	5’ – CCAGCAGCCGAATGATGTTTG– 3’
